# Barcoding Notch signaling in the developing brain

**DOI:** 10.1242/dev.203102

**Published:** 2024-12-20

**Authors:** Abigail M. Siniscalco, Roshan Priyarangana Perera, Jessie E. Greenslade, Hemagowri Veeravenkatasubramanian, Aiden Masters, Hannah M. Doll, Bushra Raj

**Affiliations:** ^1^ Department of Cell and Developmental Biology, University of Pennsylvania Perelman School of Medicine, Philadelphia, PA 19104, USA; ^2^ Institute for Regenerative Medicine, University of Pennsylvania Perelman School of Medicine, Philadelphia, PA 19104, USA

**Keywords:** CRISPR, Notch signaling, Barcoding, Brain development, Brain scRNA-seq, Neurogenesis

## Abstract

Developmental signaling inputs are fundamental for shaping cell fates and behavior. However, traditional fluorescent-based signaling reporters have limitations in scalability and molecular resolution of cell types. We present SABER-seq, a CRISPR-Cas molecular recorder that stores transient developmental signaling cues as permanent mutations in cellular genomes for deconstruction at later stages via single-cell transcriptomics. We applied SABER-seq to record Notch signaling in developing zebrafish brains. SABER-seq has two components: a signaling sensor and a barcode recorder. The sensor activates Cas9 in a Notch-dependent manner with inducible control, while the recorder obtains mutations in ancestral cells where Notch is active. We combine SABER-seq with an expanded juvenile brain atlas to identify cell types derived from Notch-active founders. Our data reveal rare examples where differential Notch activities in ancestral progenitors are detected in terminally differentiated neuronal subtypes. SABER-seq is a novel platform for rapid, scalable and high-resolution mapping of signaling activity during development.

## Teaser

**Summary:** SABER-seq is a CRISPR-Cas molecular recorder that stores transient developmental signaling inputs as permanent mutations in cellular genomes for extraction via single-cell transcriptomics.

## INTRODUCTION

Neural cell fate decisions are influenced by many factors, including the division history (lineage) of a cell, the spatial location (niche), the timing of cell birth and the signaling inputs received (Kretzschmar and Watt, 2012). Pioneering studies have shown that cellular signaling is an important mechanism for establishing the embryonic brain framework, as it regulates patterning, morphogenesis, migration, proliferation and differentiation (Cavodeassi and Houart, 2012). Classically, cellular signal transduction has been studied using signaling reporters that couple ligand or receptor activation with a downstream readout, such as fluorescent gene expression or chemical luminescence. The advantages of such reporters include identification and quantification of cells that received signaling inputs, quantification of the signaling strength and, depending on the sensitivity of the reporter, measurement of signaling dynamics. However, studies using these reporters are limited in two main aspects. First, imaging-based signaling readouts are limited by throughput, as relatively small populations of cells localized to a predefined geographical area are visualized due to time and cost constraints. Second, these methods do not provide high-resolution cell-type identification, which is especially important in highly heterogenous tissues such as the brain.

Single-cell sequencing has become the method of choice for characterizing molecular states and cell type identities with unprecedented throughput and fine resolution (Pisco et al., 2021). Recently, single-cell sequencing has been coupled with innovative genetic editing methods to characterize various biological paradigms. For example, CRISPR-Cas genome editing tools and single-cell RNA sequencing (scRNA-seq) have been combinatorially used to perform clonal tracing and lineage tree reconstruction with cell type resolution in development and disease (McKenna and Gagnon, 2019; Yao et al., 2022). Many iterations of CRISPR-based lineage recorders, also referred to as molecular recorders, have been described. One group of recorders rely on the generation of a diverse collection of edited barcodes via Cas9-induced deletions to transgenic arrays or endogenous loci (Alemany et al., 2018; Bowling et al., 2020; Chan et al., 2019; Cotterell et al., 2020; Frieda et al., 2017; Raj et al., 2018a; Spanjaard et al., 2018; Xie et al., 2023). Another group of recorders, known as ‘DNA writers’, are designed to predominantly generate small insertions to a barcode array and circumvent loss of information often observed with deletion-based Cas9 editors (Bhattarai-Kline et al., 2022; Chen et al., 2024; Choi et al., 2022; Li et al., 2023; Loveless et al., 2021, 2024; Sheth et al., 2017; Shipman et al., 2017). A third group uses orthogonal Cas proteins, such as Cas12a, for multiplexed event recordings (Kempton et al., 2022; Takasugi et al., 2022). In addition to lineage analysis, studies have applied such molecular recorders to record cellular events, such as gene expression and cellular stimuli. They have also been used in technical applications, e.g. to record videos and text messages (Chen et al., 2024; Shipman et al., 2017). However, all these uses have either been limited to cell culture systems or to prokaryotic cells, or have not been combined with scRNA-seq. Biological applications of molecular recorders using *in vivo* animal models have been restricted to cell lineage and clonal analyses.

We reasoned that CRISPR-Cas molecular recorders can be adapted to investigate a new paradigm – signaling inputs during animal development. Signaling recorders would be most beneficial where throughput and high molecular resolutions at relatively low costs are desired in an experimental system. A simple design for a signaling recorder would need to achieve three goals. First, the addition of barcode tags to cells receiving the signal input of interest. If the initial pool of labeled cells includes progenitors, the tags would propagate to later generations as the progenitor cells divide and differentiate. As such, the barcode tags represent a binary measurement (on/off) of whether the signal was active at any point during the developmental ancestry of the cell. Second, the recording system must be active long enough to enable tagging of all cells receiving signaling inputs within a developmental window. Third, the barcode tags should be retrievable via single-cell sequencing. Thus, signaling histories of cell populations can be coupled with additional measurements, such as transcriptional landscapes, chromatin accessibility and epigenetics, to provide multi-modal evaluation of developmental changes with unbiased and fine molecular resolution.

Here, we describe a new method, SABER (signal-activated barcode editing recorder), where signaling-induced activation of Cas9 promotes *in vivo* barcode editing and provides a binary measurement of signal activation. In a proof-of-principle study, we apply SABER to capture Notch signaling during brain organogenesis in zebrafish. We focused on the Notch pathway, since it is crucial for maintaining neural progenitor populations where it is widely active, thereby facilitating assessment of barcode editing efficiencies. By coupling SABER to scRNA-seq (SABER-seq), we identify brain cell types that are derived from Notch-stimulated progenitor cells. Compared to traditional signaling reporters, SABER enhances cell type resolution and enables permanent genomic recording of transient signaling events to be recovered at later timepoints. The latter provides a means to correlate signal transduction in early ancestors with terminal cell identities adopted at later stages. SABER has been designed to be modular and thus we expect that the various genetic components can be swapped to study any signaling event at any timepoint in any organism or *in vitro* system of interest. SABER belongs to a growing generation of tools where molecular recording and single-cell profiling are integrated to provide detailed molecular insight into animal development.

## RESULTS

### *tp1* enhancer-driven Notch signal sensor

**Fig. 1.**
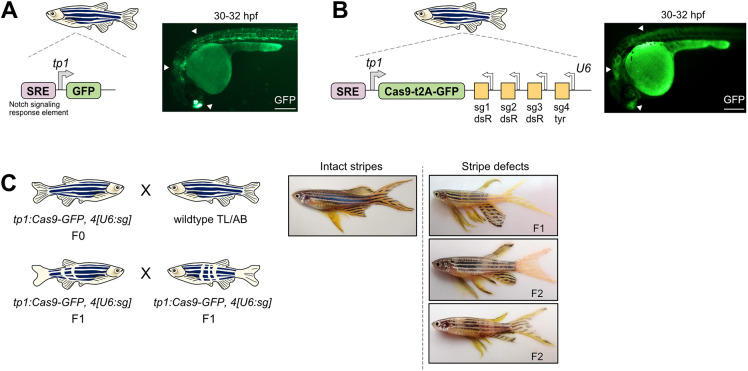
**A Notch-dependent Cas9 zebrafish transgenic reporter.** (A) Left, schematic of *tp1:GFP* transgenic reporter. Right, fluorescence image of a *tp1:GFP* zebrafish embryo at 30-32 hpf. Arrowheads highlight telencephalon, hindbrain and spinal cord. SRE, Notch signaling response element. Scale bar: 250 µm. (B) Left, schematic of *tp1:Cas9-GFP, 4[U6:sg]* transgenic reporter. sg, sgRNA. sg1-3 target dsRed sequences (dsR); sg4 targets *tyrosinase* sequence (tyr). Right, fluorescence image of a *tp1:Cas9-GFP, 4[U6:sg]* zebrafish embryo at 30-32 hpf. (C) Left, schematics of *tp1:Cas9-GFP, 4[U6:sg]* outcross (top) and incross (bottom). Right, whole-body images of adult zebrafish with intact stripes (wild type) and defective stripes from F1 and F2 transgenic adults.

In designing a signal-activated CRISPR-Cas recorder, we sought to ensure induction of Cas9 expression in a signal-dependent manner and subsequent recording of this signaling activity via Cas9-mediated mutations (edits) to a genomic barcode array. To test this idea, we focused on the Notch signaling pathway, since it is fundamental for many aspects of development, including neurogenesis, where one of its functions is to promote progenitor cell maintenance and prevent precocious neuronal differentiation (Engler et al., 2018; Lasky and Wu, 2005). Furthermore, since it is a well-characterized pathway, numerous Notch-responsive cis-elements and small molecule modulators of Notch signaling have been described in zebrafish, which we leveraged in the design of our study. The *tp1* enhancer element, comprising 12 copies of Notch co-factor binding sites (signaling response element) upstream of a minimal promoter, has been shown to be Notch responsive in mammals and zebrafish (Kato et al., 1997; Kohyama et al., 2005; Parsons et al., 2009). Moreover, it is active in Notch-responsive tissues, such as the central nervous system, pancreas, vasculature, liver and intestine (Parsons et al., 2009). We generated transgenic zebrafish with a *tp1* enhancer driving Cas9-GFP expression and U6 promoters driving constitutive zygotic expression of four sgRNAs (*tp1:Cas9-GFP, 4[U6:sg]*). Comparison of *tp1:GFP* and *tp1:Cas9-GFP, 4[U6:sg]* transgenic lines revealed similar GFP expression patterns, with fluorescence detected in the forebrain, hindbrain, spinal cord and vasculature, among other regions (Fig. 1A,B). However, we observed that *tp1:Cas9-GFP, 4[U6:sg]* transgenic fish had weaker GFP expression, likely due to the large size of the transgenic construct, and GFP expression was barely detectable beyond 48-72 h post fertilization (hpf). We observed similar Cas9-GFP silencing in other transgenic lines [e.g. *b-actin* promoter (Olactb)-driven Cas9-GFP]. This suggested that widespread and constitutive *in vivo* expression of Cas9 may not be well tolerated in developing zebrafish. To rapidly screen our transgenics for functional Cas9 activation and editing capacity, we designed one of the sgRNAs to target the *tyrosinase* (*tyr*) gene, which is necessary for pigment formation. We outcrossed *tp1:Cas9-GFP, 4[U6:sg]* founders to wild-type zebrafish and observed that F1 adults had prominent stripe disruptions in mosaic patterns (Fig. 1C). Indeed, *tp1* promoter activity has been detected in neural crest cells (Quillien et al., 2014), which give rise to pigment cells. Furthermore, Notch signaling has been implicated in pigment cell survival and adult zebrafish stripe patterning (Hamada et al., 2014). F1 adults generated from outcrossing *Olactb:Cas9-GFP, 4[U6:sg]* founders had more extensive pigmentation disruption, including near-complete loss of eye pigmentation, since the *b-actin* promoter is ubiquitously active and not Notch restricted (Fig. S1). Incrossing *tp1:Cas9-GFP, 4[U6:sg]* F1s resulted in F2 adults with mosaic stripe defects in patterns distinct from either F1 parent (Fig. 1C), suggesting *tp1* drives early Cas9 expression in somatic cells and the transgene is active across generations. Collectively, these results demonstrate that CRISPR-Cas editing can be used for recording signaling histories during zebrafish development.

### SABER records Notch signaling in the developing zebrafish brain

**Fig. 2.**
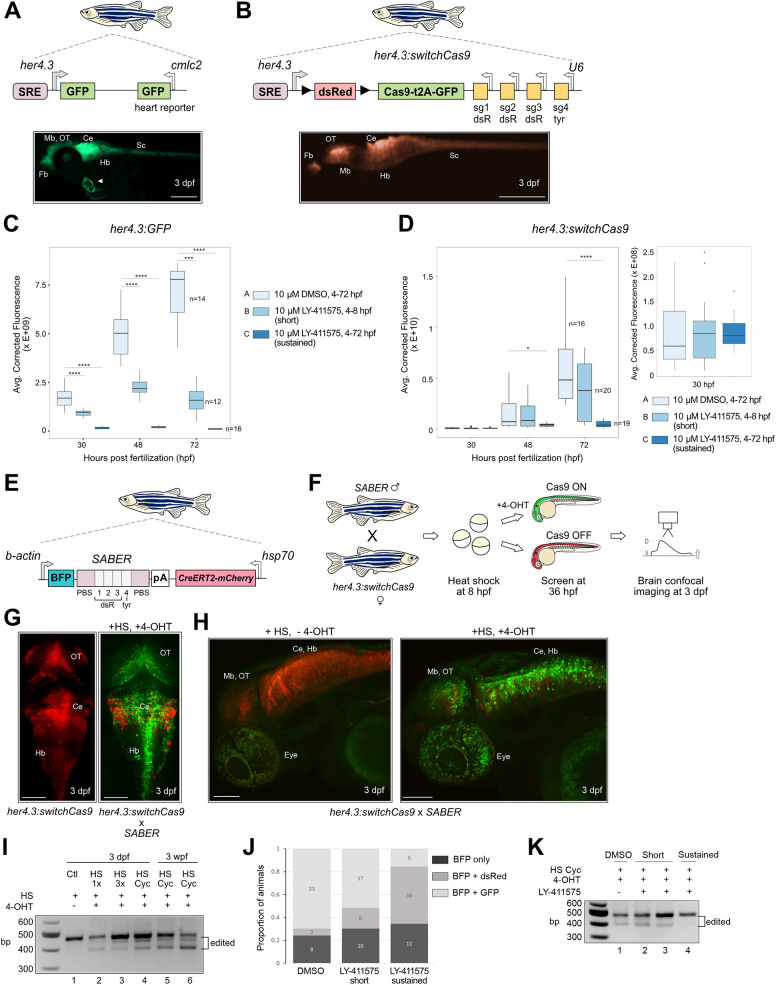
**SABER: a novel CRISPR-Cas9 signal recorder.** (A) Top, schematic of the *her4.3:GFP, cmlc2:GFP* transgenic reporter. Bottom, fluorescence image of a *her4.3:GFP* zebrafish embryo at 3 dpf. Arrowhead indicates heart GFP expression driven by *cmlc2*. Scale bar: 250 µm. Fb, forebrain; OT, optic tectum; Mb, midbrain; Ce, cerebellum; Hb, hindbrain; Sc, spinal cord. (B) Top, schematic of the *her4.3:switchCas9* transgenic reporter. The sgRNA cassette is identical to that in Fig. 1B. Bottom, fluorescence image of a *her4.3:switchCas9* zebrafish embryo at 3 dpf. Scale bar: 250 µm. (C) GFP fluorescence intensity in *her4.3:GFP* embryos after Notch inhibition. Embryos were treated with DMSO control at 4-72 hpf, LY-411575 inhibitor at 4-8 hpf (short pulse) and LY-411575 inhibitor at 4-72 hpf (sustained). Images were taken at 30, 48 and 72 hpf. *n*, number of embryos. ***P<0.001; ****P<0.0001 (Mann–Whitney *U*-test). Boxes represent the IQR; whiskers represent 1.5xIQR; dots represent outliers. (D) dsRed fluorescence intensity in *her4.3:switchCas9GFP* embryos after Notch inhibition. Right panel shows zoomed and rescaled graphs of dsRed intensity at 30 hpf. Identical treatment to the embryos in C. *P<0.05, ****P<0.0001 (Mann–Whitney *U*-test). (E) Schematic of the *SABER* Notch transgenic recorder. BFP, blue fluorescent protein; PBS, primer binding sites in purple; Grey boxes 1-4, target sites matching sg1-sg4 sgRNAs on *her4.3:switchCas9* transgene (3 dsR sites and 1 *tyr* site); *hsp70*, heat shock promoter. (F) Schematic of SABER Notch recording. A *SABER* F1 male is crossed to a *her4.3:switchCas9* F1 female. F2 embryos are heat shocked at 8 hpf to induce CreERT2 expression. A subset of embryos is treated with 4 hydroxytamoxifen (4-OHT) after heat shock until 3 dpf. Control embryos are not treated with 4-OHT. Confocal images are taken at 3 dpf. D, dorsal. V, ventral. (G) Maximum intensity projections showing the dorsal brain confocal images of a 3 dpf *her4.3:switchCas9* transgenic embryo (left) and a *her4.3:switchCas9*×*SABER* double transgenic embryo after heat shock and 4-OHT addition (right). Scale bars: 100 µm. (H) Maximum intensity projections showing lateral brain and eye confocal images of *her4.3:switchCas9*×*SABER* double transgenic embryos at 3 dpf after heat-shock cycling without (left) and with (right) 4-OHT addition. Scale bars: 100 µm. (I) Genomic DNA amplification to assess SABER barcode editing efficiency. HS, heat shock; 1×, one heat-shock pulse; 3×, three heat-shock pulses; HS Cyc, heat-shock cycling; wpf, weeks post-fertilization; bp, base pairs. (J) Proportion of 3 dpf animals from a *her4.3:switchCas9*×*SABER* cross with BFP, dsRed or GFP fluorescence after Notch inhibition. BFP only, *SABER*^+^; BFP+dsRed, *SABER*^+^ and *her4.3:switchCas9*^+^ but no GFP detected; BFP+GFP, *SABER*^+^ and *her4.3:switchCas9*^+^ and GFP detected. Numbers inside bars represent animal counts in each group. (K) Genomic DNA amplification of SABER barcodes at 3 dpf to assess editing efficiency after Notch inhibition.

To adapt CRISPR-Cas for Notch signal recording in the nervous system, we designed a new transgenic construct. Zebrafish *her4.3* (orthologous to mammalian *Hes5*) is highly expressed in neural progenitor cells and is a direct target of Notch signaling (Takke et al., 1999). A 3.4 kb promoter fragment of *her4.3* containing five Notch co-factor binding sites (signaling response element) has been established as a Notch activity reporter in multiple studies (Conner et al., 2014; Grotek et al., 2013; McCallum et al., 2020; Yeo et al., 2007). We generated transgenic zebrafish with a *her4.3* promoter driving dsRed and Cas9-GFP expression, and *U6* promoters driving constitutive expression of four sgRNAs (the same sgRNA sequences as found in the aforementioned *tp1* and *Olactb* constructs). This transgene, *her4.3:switchCas9*, represents a ‘Notch sensor’. To circumvent early silencing of transgenic Cas9 expression from constitutive promoters as discussed above, we flanked the dsRed sequence and a stop codon with *loxP* sites, such that Cas9-GFP will be ‘switched on’ when both Notch signaling and CreERT2 recombinase expression occur. This double inducible system enables recording of Notch signaling at timepoints of interest. Comparison of *her4.3:GFP* and *her4.3:switchCas9* transgenic lines revealed similar expression patterns, with fluorescence readily detected in the central nervous system (Fig. 2A,B). To confirm that *her4.3:switchCas9* transgenic animals respond to Notch activity levels, we inhibited Notch signaling using the gamma secretase inhibitor LY-411575 (Alunni et al., 2013; McCallum et al., 2020; Rothenaigner et al., 2011). We performed an early, short-pulse inhibition (4 h treatment, 4-8 hpf) before the onset of *her4.3* expression at ∼7.5-8 hpf (Takke et al., 1999), as well as sustained inhibition (4-72 hpf). Sustained Notch inhibition resulted in reduced fluorescence by 48 hpf in both *her4.3:GFP* and *her4.3:switchCas9* transgenics, with the greatest effect observed by 72 hpf (Fig. 2C,D). Short-pulse inhibition resulted in decreased fluorescence in the *her4.3:GFP* transgenic but not in the *her4.3:switchCas9* transgenic. We note that dsRed expression is not readily detectable in *her4.3:switchCas9* embryos until 48 hpf, whereas *her4.3:GFP* embryos display strong GFP expression by 24 hpf. Differences in the fluorophores used and the larger construct size of *her4.3:switchCas9* likely account for differences in relative intensities and the timing of expression. In contrast, performing Notch inhibition in *Olactb:SpCas9-GFP, 4[U6:sg]* transgenic embryos did not reduce GFP expression (Fig. S2A), demonstrating that *her4.3:GFP* and *her4.3:switchCas9* transgenics specifically respond to Notch activity, and confirming their functions as sensors.

To enable barcode recording, we designed a new transgenic construct with a *b-actin* promoter driving expression of blue fluorescent protein with a barcode array embedded in the 3′ UTR and heat-shock inducible expression of CreERT2 recombinase (Fig. 2E). This transgene represents the ‘Notch recorder’, henceforth referred to as *SABER*. The barcode is a concatemerized array of four target sites matching the four sgRNAs present on the *her4.3:switchCas9* construct. To test Notch-dependent activation of Cas9, we crossed *SABER* and *her4.3:switchCas9* transgenics, heat shocked the embryos at 8 hpf and added 4-hydroxytamoxifen (4-OHT) to a subset to trigger CreERT2-mediated *loxP* excision of the dsRed cassette and stop codon, allowing Cas9 expression (Fig. 2F). Starting at 36 hpf, we screened for GFP expression, indicating successful dsRed excision and Cas9 expression, and performed confocal imaging at 3 days post-fertilization (dpf). As expected, *her4.3:switchCas9* embryos displayed red fluorescence (Fig. 2G). In contrast, *SABER* and *her4.3:switchCas9* double transgenics had extensive conversion and GFP expression in the brain. We observed the highest GFP expression around ventricular zones of the telencephalon, optic tectum, cerebellum and rostral hindbrain (Fig. 2G). We also observed GFP expression in the retina (Fig. 2H). Raising double transgenic embryos to adults confirmed the presence of stripe defects, indicating successful Cas9 activation and function (Fig. S2B). However, the defects were more subtle than those observed in *tp1:Cas9-GFP, 4[U6:sg]* adults (Fig. 1C). This is likely due to the more restricted expression pattern of the *her4.3* promoter relative to the *tp1* promoter. Although *her4.3* is most enriched in the central nervous system, its expression has also been detected in non-neural tissues (Grotek et al., 2013; McCallum et al., 2020; Pasini et al., 2004; Takke et al., 1999). Indeed, scRNA-seq data from a published zebrafish developmental atlas (Sur et al., 2023) confirmed transient expression of *her4.3* in the xanthophore pigment lineage (Fig. S2C). To rule out the effects of leakiness of the heat-shock promoter driving CreERT2, we compared brains from *SABER*×*her4.3:switchCas9* crosses, where all embryos were heat shocked but only a subset had 4-OHT added. In the absence of 4-OHT, we observed, at most, one or two cells that were GFP positive, confirming that Notch, CreERT2 and 4-OHT are all required for activation of Cas9 (Fig. 2H).

### Calibration of SABER recording

Next, we determined if Notch-dependent Cas9 activation results in barcode editing. We extracted genomic DNA (gDNA) from the heads of 3 dpf double transgenic (*her4.3switchCas9^+^* and *SABER^+^*) embryos and PCR amplified SABER barcodes. We observed no editing in animals that were heat shocked but were not treated with 4-OHT, whereas animals treated with heat shock and 4-OHT displayed shifts in band sizes, indicating editing (Fig. 2I). To calibrate the recording period and editing levels, we tested various heat shock conditions. We observed that a single 35 min heat-shock pulse at 8 hpf (Fig. 2F) resulted in a low level of editing (Fig. 2I, 1× HS). Increasing to 3×35 min pulses at 8, 24 and 32 hpf resulted in minor differences in band intensities when observed on a gel (Fig. 2I, 3× HS). Next, we tested heat shock cycling where embryos were subject to 17 heat-shock pulses from 8 to 48 hpf (Fig. S2D). This resulted in enhanced barcode editing (Fig. 2I, HS Cyc) with no significant effect on mortality rates (Fig. S2E). We observed that Cas9-GFP expression slowly faded 4-5 days after it was first activated (Fig. S2F), suggesting that signaling can be recorded for a window of ∼96-120 h after the initiation of heat shock and 4-OHT addition. Furthermore, we extracted gDNA from the brains of 3 weeks post-fertilization (3 wpf) double transgenic animals that were subjected to heat-shock cycling as embryos and we observed strong barcode editing (Fig. 2I). This demonstrates that signaling histories are permanently recorded and can be extracted long after the recording period has ended. To further confirm editing occurs only in the presence of active Cas9-sgRNA complexes, we extracted gDNA from the heads of 3 dpf progenies of *SABER*×*her4.3:GFP* (no Cas9 or sgRNA cassette) and *SABER*×*her4.3:loxP-dsRed-loxP-Cas9-GFP* (no sgRNA cassette) crosses following heat-shock cycling. We observed no barcode editing when Cas9 and/or sgRNAs are not expressed *in vivo* (Fig. S2G,H). Finally, we tested if signal recording occurs when Notch activity is inhibited. Performing a short pulse inhibition in embryos from a *SABER*×*her4.3:switchCas9* cross resulted in a slight reduction in the number of embryos with detectable GFP expression and no noticeable difference in barcode editing relative to DMSO controls (Fig. 2J,K). In contrast, sustained Notch inhibition resulted in a dramatic reduction in the number of embryos expressing GFP and suppressed barcode editing (Fig. 2J,K). Collectively, these results confirm that SABER can specifically and permanently record Notch signals as edited barcodes via inducible Cas9 activity.

### Validation of SABER recording specificity

**Fig. 3.**
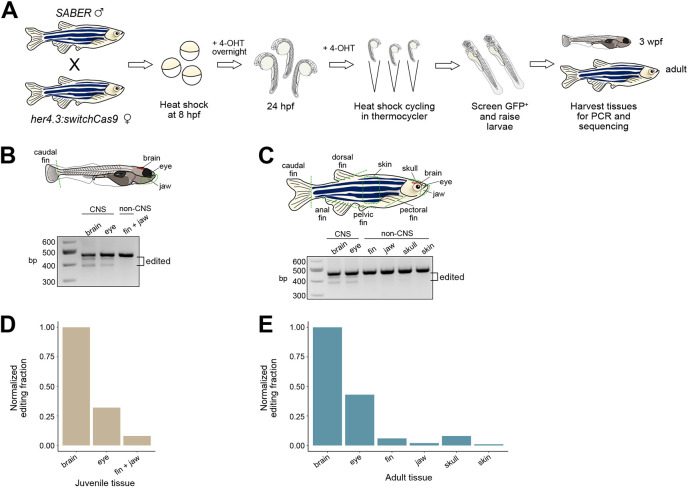
**SABER barcode editing is enriched in central nervous system tissues.** (A) Schematic of SABER recording protocol. (B,C) Genomic DNA amplification of SABER barcodes from (B) 3 wpf fish and (C) adult fish tissues. CNS, central nervous system. Dotted green lines represent dissected regions. (D,E) Fractions of edited SABER barcodes in various tissues normalized to brain tissue from (D) 3 wpf fish and (E) adult fish.

To compare the levels of editing across various tissues, we performed Notch recording with heat-shock cycling as described above, and subsequently extracted gDNA for PCR analysis and barcode sequencing from 3 wpf juvenile and 1-year-old adult fish (Fig. 3A). We isolated gDNA from the brain, eye, caudal fin and jaw tissues of 3 wpf double transgenic fish. We isolated gDNA from the fins, skin, jaw, skull, brain and eye tissues of adult double transgenic fish. As expected, we observed shifts in barcode size in central nervous system tissues by PCR (Fig. 3B,C). Sequencing SABER barcodes from the isolated tissues confirmed that the brain had the most edited barcodes followed by the eye (Fig. 3D,E). Since the eye comprises auxiliary structures not derived from the retina, this mixed tissue composition likely contributes to the lower edited fractions relative to the brain. The remaining non-neural tissues had the lowest edited fractions in both juvenile and adult stages. For example, the adult skin had a 99% decrease in the edited barcode fraction compared to the adult brain. In conclusion, our findings validate that Cas9-mediated barcode editing is enriched in central nervous system tissues where the *her4.3* promoter is activated and sustained by Notch signaling.

### Combining signal recording with scRNA-seq

**Fig. 4.**
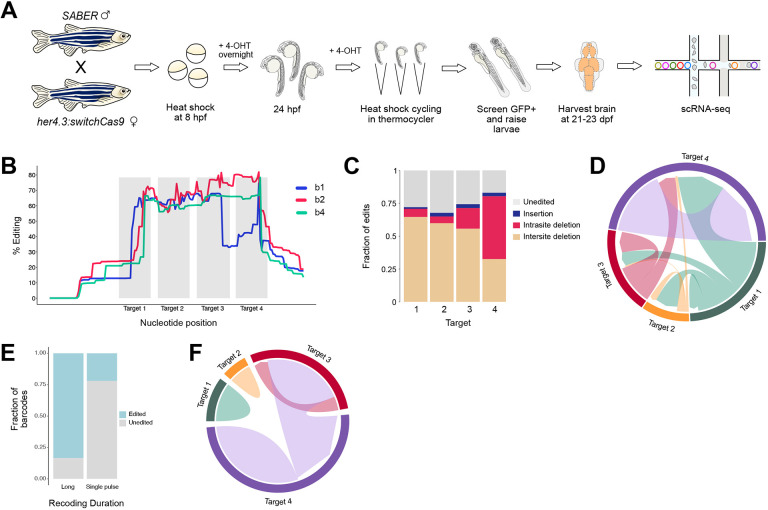
**SABER-seq recovers Notch-edited barcodes from single cell transcriptomes.** (A) Schematic of SABER-seq experimental protocol. (B) Percentage editing across each nucleotide in SABER-seq barcodes. Positions of the four CRISPR target sites are in gray. Data are from three brains. Only deletion mutations are shown. (C) Edit type at each target site. (D) Chord diagram of the nature and frequency of deletions. Each colored sector represents a target site. Links between target sites indicate inter-site deletions; self-links indicate intra-site deletions. Link widths are proportional to edit frequencies. (E) Fraction of barcodes that are edited following long- versus single-pulse duration of Notch recording. (F) Chord diagram of the nature and frequency of deletions after one heat-shock cycle.

Next, we performed Notch recording experiments and analyzed SABER barcodes by scRNA-seq of whole brains from 21-23 dpf juveniles using the 10x Chromium platform (Fig. 4A). This method, SABER-seq, enables simultaneous recovery of endogenous mRNAs and SABER barcode transcripts. We prepared SABER libraries via PCR amplification of barcode cDNAs and sequenced the libraries separately from corresponding transcriptome libraries. In our first iteration of SABER-seq, we recovered barcodes from 3454 cells from two brains (b1 and b2). This represents 6-8% recovery from all profiled cells per animal (version referred to as SABER_v0). To improve recovery, we optimized SABER-seq library preparation steps (see Materials and Methods) and recovered 3156 cells from a third brain (b4), which represents 22% recovery (version referred to as SABER_v1). In summary, we obtained 6610 barcodes from single cells, of which 5351 barcodes (81%) were mappable to the unedited reference sequence, highlighting the scalability of this approach (Table S1). The remaining barcodes were too short (less than 50 bp) for unambiguous mapping. Large deletions resulting in very short amplicons have been previously described as a limitation of barcode arrays with tandem sgRNA design (Salvador-Martínez et al., 2019). To analyze the spectrum of mutations, we considered only the mappable cohort of barcodes for downstream analyses. As expected, barcode editing was highest around the sgRNA target sites but extended upstream and downstream of the targets, consistent with larger deletions (Fig. 4B). Furthermore, 83.4% of barcodes had edits in at least one target site, confirming effective Cas9 expression and activity in Notch-induced cells. Deletions were the predominant mutations and could be categorized as intra-site, which represent edits within a target, or inter-site, which represent edits that span two or more targets or extend outside the sgRNA loci (Fig. 4C,D). The frequency of each type of deletion varied among the four target sites with deletions spanning target sites 1 to 4 being the most frequent (Fig. 4D). To test if the fraction of edited barcodes changes with recording duration, we sequenced SABER barcodes from the brain of a 21 dpf juvenile that was subjected to only a single pulse of heat shock and 4-OHT at 8 hpf. We observed 22% barcode editing with one heat shock pulse (Fig. 4E). Notably, the nature of the edits also varied with more intra-site versus inter-site deletions (Fig. 4F). Thus, smaller deletions are observed when recording over a shorter induction window. Collectively, these results demonstrate that the timing and duration of Notch recording can be varied to tag Notch-active cells sparsely or comprehensively, depending on the goals of the experiment.

### A refined zebrafish whole brain atlas

**Fig. 5.**
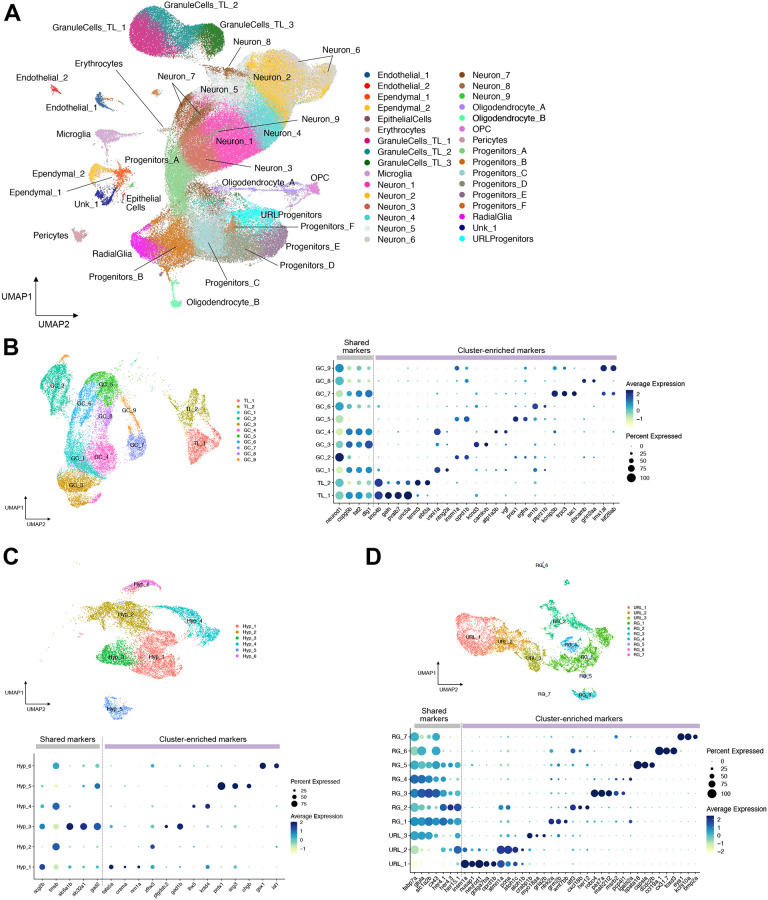
**An expanded juvenile zebrafish brain atlas.** (A) UMAP embedding of 148,853 cells showing 32 clusters resulting from coarse-grained clustering. (B-D) UMAP embedding and dot plots of marker gene expression from subclustering of (B) granule cells and torus longitudinalis cells, (C) hypothalamus cells, and (D) radial glia and upper rhombic lip progenitor cells.

To superimpose Notch signaling histories captured via SABER-seq onto finely resolved brain cell types and cell states, we first established a juvenile zebrafish brain cell catalog. In previous work (Raj et al., 2018a), we used inDrops to describe brain cell taxonomy from similar aged animals (23-25 dpf) but to avoid technical artifacts of mapping SABER-seq data obtained via the 10x Chromium platform onto pre-existing inDrops data, we collected new samples. We profiled brain regions (forebrain, diencephalon+midbrain, and hindbrain tissues) and whole brains (including brains b1, b2 and b4 from SABER-seq experiments) from 21-23 dpf animals using 10x Chromium. We sequenced 156,353 cells and retained 148,853 cells for downstream analysis after quality filtering. Using a combination of initial coarse-grained computational clustering and subsequent rounds of iterative clustering, we classified 137 neuronal, non-neuronal and progenitor cell subtypes and cell states (Fig. 5, Figs S3 and S4, Tables S2 and S3). This represents more annotations than our previous dataset (Raj et al., 2018a) (137 versus 105), which is expected since we sequenced greater than twice the number of cells. For example, we identified nine granule cell subclusters, 11 pallium subclusters, eight subpallium subclusters, seven hypothalamus subclusters, nine dorsal habenula subclusters, six ventral habenula subclusters, and seven radial glia subclusters, among many others. Although we used classical markers from literature to assign subtypes to anatomical regions, as described previously (Raj et al., 2018a, 2020), a few cell types could not be confidently mapped to discrete regions and were labeled by their enriched cluster marker only (e.g. Neuron_mef2cb^+^). There were also two clusters whose identities we could not fully resolve (Unk_1, Unk_2). We observed overlap between markers identified in our study and those used to classify and *in vivo* validate subtypes described in recent scRNA-seq studies, where corresponding brain subregions were dissected for profiling. For example, a scRNA-seq study (Pandey et al., 2023) of pallium (*eomesa*, *bhlhe22*, *zbtb18* and *emx3* expressing) and subpallium (*dlx2a*, *dlx5a* and *dlx6a* expressing) tissues identified cells expressing *c1ql3b* (Pal_2), *lhx9* (Pal_3), *pvalb7* (Pal_10), *lhx6a* and *lhx8a* (SubPal_4), and *six3b* (SubPal_6) (Fig. S4C,E). A scRNA-seq study of *gng8^+^* habenula cells (Pandey et al., 2018) identified cells expressing *wnt7aa* (cluster DHab_4), *adcyap1a* (Dhab_5), *foxa1* (Dhab_6), and *cbln2b* (Dhab_8) (Fig. S4F). In addition, a scRNA-seq study of the preoptic area and hypothalamus (Shafer et al., 2021) identified cells expressing *trh* (POA_2), *prdx1* (Hyp_5), and *gsx1* (Hyp_6) (Fig. 5C). Finally, a scRNA-seq study (Mitic et al., 2024) of radial glia [*fabp7a*, *glula* and *gja1b* (previously known as *cx43*) expressing] identified cells expressing high levels of *her4.3* (RG_2) and *robo4* (RG_3) (Fig. 5D). Collectively, we observe that our whole-brain dataset contains fewer subtypes than those obtained from enriched profiling of individual brain subregions. This is expected, as we have less than 1% coverage of 21-23 dpf brain cells (there are estimated to be more than 1 million cells in the zebrafish brain at this stage). Nevertheless, we identified multiple rare populations, including pineal cells (56 cells, representing 0.04% of dataset) and olfactory bulb cells (80 cells, representing 0.05% of dataset) (Fig. S4H, Table S2). The dataset can be explored using the accompanying app available at https://github.com/BushraRaj-Lab/ZBrainAtlas_Siniscalco.

### Signal tracing confirms that Notch stimulated progenitors give rise to the majority of zebrafish brain cell types

**Fig. 6.**
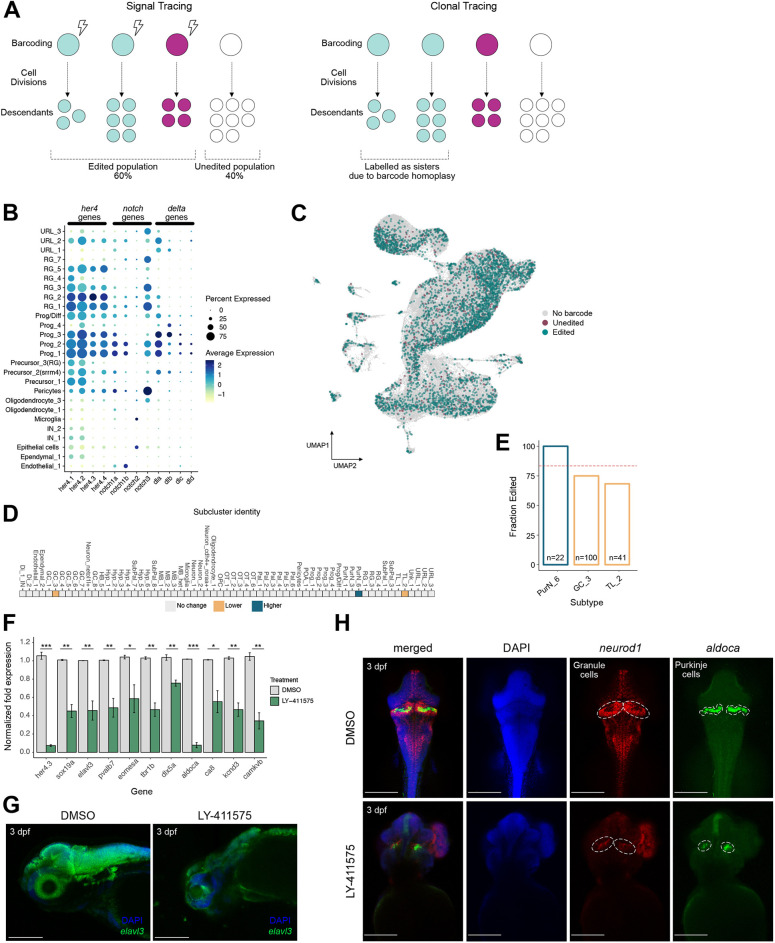
**SABER-seq barcodes matched to cell type atlas.** (A) Schematic of signal tracing versus clonal tracing. (B) Gene expression dot plot of *her4* variants, Notch genes and Delta genes in the indicated subclusters. (C) UMAP embedding of 148,853 cells from Fig. 5A. Cells from which SABER barcodes were recovered are colored to indicate edited and unedited barcodes. (D) Identification of subclusters where the fraction of edited barcodes diverged from the overall editing frequency observed in the brain. Only subclusters where at least 20 cells had SABER barcodes were considered. Gray squares, no significant change; blue squares, higher fraction; orange square, lower fraction. *P*<0.05 (binomial test). (E) Fraction of edited barcodes in three subclusters with significant change from overall editing rate (dotted red line) are shown. *n*, number of cells across three brain SABER-seq samples. (F) Gene expression from 3 dpf zebrafish was analyzed by RT-qPCR across samples treated with DMSO (control) and LY-411575 to inhibit Notch from 4-72 hpf. *aldoca* and *ca8* are enriched in Purkinje cells. *kcnd3* and *camkvb* are enriched in granule cell neurons. Data are mean±s.e.m. *n*=3 biological replicates. *P<0.05, **P<0.01, ***P<0.001 (unpaired two-tailed *t*-test). (G) Lateral images of HCR RNA-FISH in 3 dpf zebrafish using *elavl3* probes and stained for DAPI. Same treatment as in F. Scale bars: 200 µm. (H) Dorsal images of HCR RNA-FISH in 3 dpf zebrafish using *neurod1* and *aldoca* probes, and stained for DAPI. Subsections of the cerebellum are outlined. Same treatment as in F. Scale bars: 200 µm.

Next, we matched SABER-seq barcodes with our annotated brain cell dataset. We obtained 277 uniquely edited SABER barcodes of varying frequencies. The most abundant barcode comprised 23% profiled cells and was found in brain samples from all three animals, suggesting that barcode homoplasy (i.e. identical barcodes arising in parallel independently) occurs with SABER-seq. Barcode homoplasy has been previously reported with CRISPR-Cas lineage recorders that rely on *in vivo* expression of Cas9 in zebrafish (Raj et al., 2018a). Although barcode homoplasy confounds clonal tracing (i.e. identifying common ancestors), it is not a limitation when barcodes are used to investigate signaling histories. Our signal tracing method is designed to identify the population within a profiled sample that is derived from progenitors where detectable signaling occurred (Fig. 6A). SABER-seq barcodes overlapped 127/137 cell annotations in our dataset (Table S4). The missing cell types were rare populations that accounted for less than 0.2% of the dataset. Thus, barcodes were likely not detected in those cells due to lack of sampling depth. These results confirm that SABER-seq enables efficient barcoding of cells derived from Notch-active ancestors. We also asked whether cells of a given cell type are detected more or less often in the SABER-seq dataset relative to the rest of the dataset. We identified 21 subclusters where cells were detected more frequently and 22 subclusters where cells were detected at a lower frequency (Table S4). These results suggest some bias in the expression and/or amplification of the Notch recorder transgene, which is driven by a *beta-actin* promoter, among the different cell types.

Since Cas9 expression was initiated in cells expressing *her4.3:switchCas9* upon Notch activation, edited SABER barcodes should be detected in the progeny of Notch stimulated-*her4.3^+^* ancestor cells, effectively acting as a permanent tracer of descendants. The zebrafish *her4* gene cluster is organized as tandem duplicate repeats (*her4.1*-*her4.5*) on chromosome 23, with nearly identical transcripts except for some polymorphisms in the 3′ UTR. Additionally, all variants are translated into identical peptides. We inspected the expression of endogenous *her4* variants in our dataset. We observed highest expression of *her4* variants, including *her4.3* in several clusters of radial glia, which have neural stem cell properties, and other neural progenitor and precursor cell clusters in our dataset at 21-23 dpf (Fig. 6B, Fig. S5). This is consistent with the constitutive neurogenic capacity of these cells at all stages from embryo to adult (Schmidt et al., 2013). Furthermore, these cell types give rise to all neuronal subtypes in the zebrafish brain (Schmidt et al., 2013). Accordingly, we detect edited SABER barcodes in neural progenitors, immature neurons and mature neurons, as expected (Fig. 6C). Interestingly, we also recovered edited SABER barcodes from several classes of differentiated non-neuronal cells, including epithelial cells, ependymal cells, oligodendrocytes and pericytes. We detect strong *her4.3* expression in pericytes in our dataset (Fig. 6B). However, *her4.3* transcripts are barely detectable in other non-neuronal cell types at 21-23 dpf. A possibility is that *her4.3* was transiently expressed during development of these cell types before being turned off during or after differentiation, similar to its expression patterns observed in the transition from neural progenitors to neurons. Consistent with this, oligodendrocytes and ependymal cells have been identified as *her4.3*-derived progeny from lineage tracing experiments using scRNA-seq (Cavone et al., 2021; Lange et al., 2020). Furthermore, *her4.3* has been detected in the hypoblast, presomitic mesoderm and neural crest cells during development, and in blastema cells during fin regeneration, highlighting transient non-neuronal expression domains (Grotek et al., 2013; McCallum et al., 2020; Takke et al., 1999). Notably, Notch signaling has been shown to be important for cell fate specification of the aforementioned non-neuronal cell types in development and regeneration (Carlén et al., 2009; Kim et al., 2008, 2020; Wang et al., 2011, 2014). Regulation of *her4.3* expression is orchestrated via *notch1a*, *notch1b* and *notch3* receptor genes (Yeo et al., 2007). Indeed, expression of at least one of these notch genes was detected in cell types expressing *her4.3*, with *notch3* showing the highest levels and percentage expression in our dataset (Fig. 6B). Among Notch ligands, Delta has been implicated in Notch signaling in the zebrafish brain (Dong et al., 2012). In our dataset, *dla* had the strongest expression and overlapped with Notch genes in the cell types where *her4.3* was highly expressed. Collectively, our results indicate that widespread Notch and *her4.3* activities in multiple cell types in the brain can be effectively captured by our technology.

### Exploring differences in barcode editing frequency between cell types

Finally, we asked whether the fraction of edited barcodes within any subcluster diverged from the overall editing frequency observed in the brain dataset. Most clusters did not show statistically significant differences in editing frequencies (Fig. 6D), suggesting that, at the time of recording, Notch signaling was active in many pools of ancestral progenitors but did not impact terminal neuron subtype fate specification. This is consistent with the early developmental role of Notch in overall maintenance of progenitor pools by inhibition of any neuronal fate decisions. Is Notch involved in cell fate specification of discrete brain neuron subtypes? Only a few studies have found that regulation of Notch signaling in vertebrate neural tissues drives generation of specific neuron cell types (Cau and Blader, 2009; Pierfelice et al., 2011). For example, Notch signaling in zebrafish spinal cord is required for generating v2 inhibitory interneurons rather than v2 excitatory interneurons (Batista et al., 2008), and for generating KA′ interneurons while suppressing primary motoneuron fate (Shin et al., 2007). Notch is also required to suppress projection neuron fates in progenitor cells expressing markers of both projection neuron and photoreceptor identities in zebrafish pineal gland in the brain (Cau et al., 2008). In our dataset, we identified two subclusters, GC_3 (granule cell subtype) and TL_2 (torus longitudinalis subtype), with lower fractions of edited barcodes than expected (Fig. 6D,E). We also identified one subcluster, PurN_6 (Purkinje cell subtype), with a higher proportion of edited barcodes (100% editing). Interestingly, another Purkinje cell subtype, PurN_2, also had 100% editing frequency (*n*=15/15 cells, *N*=3 brains) but the sample size was too small to detect statistical significance. These results suggest that GC_3 progenitors and TL_2 progenitors may have received less or delayed Notch signal during the temporal window when we recorded Notch activity. In contrast, PurN_6 progenitors may have received higher or more sustained Notch activity. In the mouse cerebellum and human cerebellar organoids, *Sox2*^+^ bi-potential embryonic progenitors can give rise to Purkinje cells and granule cells, and the decision between these fates requires Notch signaling (Zhang et al., 2021). Cells where Notch remains active generate Purkinje cells, whereas cells where Notch is turned off generate granule cells. Although a similar mechanism has not been validated in zebrafish, our data are consistent with Notch activity potentially positively impacting the adoption of Purkinje cell fate.

We pharmacologically inhibited Notch signaling from 4-72 hpf, which overlaps the time window used for recording Notch activity in SABER-seq experiments and assessed effects on neuronal populations at 3 dpf. We confirmed suppression of Notch activity by decreased *her4.3* mRNA expression relative to DMSO-treated animals via qPCR (Fig. 6F). Loss of Notch activity resulted in decreased expression of the radial glia marker *sox19a*, as well as of various neuronal markers, including the broad neuronal marker *elavl3*, suggesting that early and prolonged Notch inhibition impairs overall neurogenesis by 3 dpf (Fig. 6F, Fig. S6). HCR RNA-FISH using *elavl3* probes confirmed decreased neuronal populations (Fig. 6G). This agrees with previous studies showing that loss of developmental Notch signaling results in deficits in secondary neurogenesis (i.e. late-differentiating neuron populations born after 2 dpf) due to depletion of neuroepithelial progenitor cells (Bingham et al., 2003; Jiang et al., 1996). Neuroepithelial progenitors give rise to radial glia cells that are responsible for neuronal subtype expansion at later stages (Alunni et al., 2020). Next, we performed HCR RNA-FISH using markers for Purkinje cells (*aldoca*) and granule cell (*neurod1*) neurons in Notch inhibited and control animals. The population sizes of both subtypes appeared reduced relative to control, corroborating the qPCR results (Fig. 6H). However, gross brain morphology impairments precluded quantification of differences. Since both these cerebellar cell types are born after 48 hpf, it is difficult to resolve whether loss of Notch signaling affects one subtype more than the other in our inhibition assay due to widespread effects on secondary neuron populations when Notch is suppressed during early development. In summary, results from the Notch inhibition experiments together with our findings from the SABER-seq data support a general role for Notch signaling in progenitor maintenance in early development. However, whether Notch directly regulates Purkinje cell or granule cell fate requires further testing. Collectively, our results demonstrate the power and sensitivity of SABER-seq for converting transient signaling events to permanent traceable marks and for identification of cells whose fates are determined downstream of these signaling activities.

## DISCUSSION

In the past few years, DNA molecular recorders have rapidly become one of the leading methods for large-scale parallel recording of various cellular paradigms, owing to their scalability and compatibility with genomics, particularly scRNA-seq. Here, we have developed a molecular recorder, SABER, and applied it to Notch signal tracing during zebrafish brain development. We designed SABER to barcode cells that are activated by a signal of interest, calibrated the recording period to maximize cell barcoding and made the system compatible with scRNA-seq. We chose to record Notch activity in this proof-of-principle study, since Notch is widely active in neural progenitor and stem cells of the brain. This facilitates benchmarking barcode editing frequencies and inheritance in our brain scRNA-seq datasets, as we expect nearly all neural cell types to contain edited barcodes in their transcriptomes. The power of SABER-seq rests on engineering transient Notch cellular signals to trigger permanent CRISPR/Cas-mediated edits in a genomic transgenic array, which are then propagated during cell division, enabling long-term storage of binary (on/off) recordings of signaling activity in ancestral cells. The recording is inducible and can be initiated at developmental windows of interest using 4-OHT and heat-shock treatments. Additionally, SABER-seq enables simultaneous recovery of cellular transcriptomes to identify individual cells and cell types that develop after Notch activation. We also demonstrate the modular nature of SABER-seq using two different Notch-sensitive promoters, *tp1* and *her4.3* in zebrafish, which were conveniently swapped via Gateway cloning. We expect that SABER-seq can be adapted for measuring additional signaling pathways by using a promoter with the appropriate upstream signaling response element.

Several lines of evidence in our data and published studies highlight transient expression of *her4.3* in non-neural cells. For example, *her4.3* is necessary for paraxial mesoderm segmentation during early development (Pasini et al., 2004). Additionally, Notch signaling and *her4.3* expression are activated in proliferative blastema cells during zebrafish fin regeneration (Grotek et al., 2013). Thus, the single-cell resolution provided by SABER-seq is advantageous and can be used to explore previously unrecognized sites of *her4.3* and Notch activities in various biological contexts.

In vertebrates, Notch signaling regulates neuronal diversity by controlling the timing of cell birth; it maintains progenitor populations and prevents their untimely differentiation, which can deplete progenitor pools and negatively impact brain expansion and diversification (Cau and Blader, 2009). Although ample evidence in *Drosophila* demonstrates that Notch directly regulates specification of neuronal subtype identities via asymmetric cell division and differential segregation of cell fate determinants (Bardin et al., 2004; Udolph, 2012), a similar role for Notch during vertebrate neurogenesis has been elusive. Indeed, proof of Notch signaling directly controlling the identity of discrete neuronal subtypes has only been described in a limited number of neuron types in the brain, eye and spinal cord of some vertebrate systems. Our SABER-seq results are in line with these findings. We find that most neuron subtypes in the zebrafish brain do not exhibit differential barcode editing frequencies during the early developmental window when we recorded Notch signaling, supporting a general role for Notch in progenitor maintenance. At least one Purkinje cell subtype had a higher barcode editing rate, suggesting that Notch activity may be instructive for its development, similar to findings in the mammalian cerebellum (Zhang et al., 2021). It remains to be determined whether these effects are directly regulated by Notch; however, our results demonstrate that SABER-seq can be used to explore the relationship between signaling and cell fate decisions.

SABER-seq provides several advantages over previous iterations of signaling reporters. First, since it is a sequencing-based method, it is scalable to millions of cells in a cost-effective manner. We describe its application with 10x Chromium, but we anticipate it can be combined with other lower cost scRNA-seq platforms, such as sci-seq (Martin et al., 2023), by adapting the SABER library amplification protocol. Second, the permanence of the barcodes precludes the need for real time observation of signaling activity and removes the constraint of short-term observation, issues that plague fluorescent signal recorders where fluorescence is detectable for a limited number of cell divisions. Third, it provides a detailed measurement of cellular gene expression states while fluorescent reporters lack this ability. These features make SABER-seq an attractive alternative to traditional signaling reporters when high throughput at low cost, fine resolution, long-term storage and binary measurements of signaling are desired. Nevertheless, we foresee several optimizations. First, differences in barcode expression between cell types and short amplicons resulting from large CRISPR/Cas deletions that often remove primer-binding sites introduce bottlenecks in barcode library generation (Raj et al., 2018a). To circumvent the latter, fusion proteins of Cas9 and DNA nucleotidylexotransferase (DNTT, also known as TdT), a template-independent polymerase, can be used to favor sequential insertions and enhance barcode recovery (Li et al., 2023; Loveless et al., 2021). Second, SABER-seq provides binary readouts of whether barcodes are either edited (signal detected) or unedited (no signal or insufficient signal). Due to complexities of double strand break repair outcomes, it is challenging to relate differences in signaling levels, duration or oscillations of Notch signaling with the number of individual edits on the barcode. For example, the mechanism used to repair double-strand breaks can differ between cell types and/or developmental stages (e.g. highly error-prone alternative end-joining versus less error-prone classical non-homologous end-joining) (Raj et al., 2018a; Thyme and Schier, 2016). For biological questions where more precise measurements of signaling levels within specific cell types are desired, follow up experiments can be performed using fluorescence or chemical signaling reporters, along with cell-type markers identified by SABER-seq. Finally, scRNA-seq results in tissue destruction and loss of spatial context. Although it is possible to perform in silico mapping of cells to their most likely location using spatial markers, it is still prediction based. To mitigate these drawbacks, we anticipate merging SABER-seq with spatial transcriptomics to preserve tissue architecture and niche information.

SABER-seq measures a different paradigm from the one measured by lineage molecular recorders. The latter are designed to determine cell division patterns and relatedness between cells. As a result, high barcode diversity and low barcode homoplasy are required for unambiguous mapping of clonal relationships. In contrast, SABER-seq is engineered to determine the fraction of a population arising from signal-activated ancestral progenitors. For signals like Notch that are active in many cells and for long temporal windows in a given tissue, barcode editing is expected in a higher fraction of the profiled cells. For signals with more-restricted spatiotemporal patterns, a lower fraction of profiled cells will display barcode editing. By varying the timing of signal recording, multiple signal tracing snapshots can be stitched together to track variations in signaling patterns throughout development via changes in the barcoded cell populations.

We discuss a few exciting applications of SABER-seq in its current format. First, it can be combined with genetic perturbations to investigate the role of signaling regulation on development. Here, signal barcoding would be carried out in control and F0 knockouts (i.e. crispants) of signaling components, and the effects on the composition of cell populations and fraction of editing across cell types would be assessed. Such ‘signalomes’ would provide a method for relatively rapid and unbiased high dimensional molecular and cellular phenotyping (Saunders et al., 2023) to identify candidate signaling effectors that impact development and subtype specification for downstream functional validations. Second, SABER-seq can be applied in non-developmental paradigms to study how signaling activities change throughout cell fate remodeling during regeneration of limbs or organs, as well as during oncogenesis and metastases. For example, although Notch signaling is activated in zebrafish fin blastema cells, it is unclear in which cell types the signals originate and how the signals impact the cell types that regenerate to re-establish the fin (Grotek et al., 2013). SABER-seq can be used to quantify the fraction of the regenerating tissue where Notch is re-activated and to identify cells that were derived from Notch-active blastema cells. Third, multiplexed single-cell profiling assays are being increasingly used with the goal of ultimately generating richly annotated ‘cell trees’, where lineage, chromatin, epigenetic and transcriptional measurements of cellular states are embedded along branches of the tree (Domcke and Shendure, 2023; McKenna and Gagnon, 2019). Since SABER-seq is compatible with existing single-cell protocols, it can be integrated with additional multiplexed measurements to further clarify key events in developmental and differentiation hierarchies.

In summary, SABER-seq lays the foundation for massively parallel signal recording with single cell resolution and establishes another use of CRISPR/Cas molecular recorders in development. We anticipate further design adaptations of this technology for multiplexed recording of two or more different signaling pathways or for multiplexed temporal recording of the same pathway using biochemically diverse orthologous Cas proteins.

## MATERIALS AND METHODS

### Zebrafish husbandry

This work was performed under protocol numbers 807110 and 807259, which were approved by the University of Pennsylvania's Office of Animal Welfare of Institutional Animal Care and Use Committee (IACUC). All zebrafish work in this study follows the University of Pennsylvania Institutional Animal Care and Use Committee regulations.

### Constructs for transgenesis

The plasmid pTol2-her4.3-eGFP-cmlc2-GFP was a gift from Laure Bally-Cuif. The plasmid pTol2-tp1bglob-eGFP (*tp1:GFP* in this study) was obtained from Addgene (plasmid 73586). The plasmid pTol2-tp1-SpCas9-t2A-GFP, 4xU6:sgRNA (*tp1:Cas9-GFP, 4[U6:sg]*) and pTol2-her4.3:loxP-dsRed-loxP-SpCas9-t2A-GFP, 4xU6:sgRNA (referred to as *her4.3:switchCas9*) were constructed as follows. Individual sgRNAs targeting sites 1-4 of the SABER array were cloned into four separate U6x:sgRNA plasmids (Addgene 64245, 64246, 64247 and 64248) as described previously (Yin et al., 2015). The U6x:sgRNAs were assembled into a contiguous sequence in the pGGDestTol2LC-4sgRNA vector (Addgene plasmid 64242) by Golden Gate ligation. The resulting 4xU6:sgRNA sequence was PCR amplified and ligated into the backbone of pDestTol2pA2-U6:gRNA (Addgene plasmid 63157) after the vector was first digested with ClaI and KpnI to generate the pDestTol2pA2-4xU6:sgRNA plasmid. The middle entry vector pME-loxP-dsRed-SV40pA-loxP-Cas9-t2A-GFP was generated by first PCR amplifying the loxP-dsRed-SV40pA-loxP sequence from the pTol2Olactb:loxP-dsR2-loxP-EGFP vector (a gift from Atsushi Kawakami, Tokyo Institute of Technology, Japan). The amplified sequence was then ligated using Gibson cloning into the backbone of plasmid pME-Cas9-t2A-GFP (Addgene plasmid 63155) after the vector was digested with SalI. The final constructs were generated via multisite Gateway with p5E-tp1 (Addgene plasmid 73585) or p5E-her4.3-GFP (a gift from Laure Bally-Cuif, Institut Pasteur, Paris, France) together with pME-Cas9-t2A-GFP (Addgene plasmid 63155) or pME-loxP-dsRed-SV40pA-loxP-Cas9-t2A-GFP, p3E-polyA (Tol2 kit) and pDestTol2pA2-4xU6:sgRNA.

The SABER barcode transgenic vector pTol2-Olactb-BFP-SABER-SV40-hsp70-mCherry-CreERT2-SV40 was constructed as follows. The SABER barcode sequence was ordered as a gene block from IDT and was cloned into a multiple cloning site (MCS) in the 3′ UTR of a BFP-coding sequence in the base vector pTol2-Olactb-BFP-MCS-SV40-hsp70-mCherry-CreERT2-SV40. Plasmids have been deposited in Addgene (https://www.addgene.org/Bushra_Raj/).

### Generation of transgenic zebrafish

All transgenic lines were generated by injecting one-cell embryos with Tol2 mRNA and corresponding plasmids, identifying founders and raising F1 adults, as described previously (Raj et al., 2018b).

### Notch recording

Male *SABER* adults were crossed to female *her4.3:switchCas9*, *her4.3:GFP* or *her4.3:loxP-dsRed-loxP-Cas9-GFP* adults. For single heat-shock treatment, 8 hpf embryos were heat shocked for 35 min at 37°C. Embryos were transferred to a Petri dish of fresh media containing 10 μM 4-OHT (4-hydroxytamoxifen) and incubated at 28.5°C until screening at 2-3 dpf. For triple heat-shock treatment, embryos were first heat shocked at 8 hpf and 4-OHT added as described above. Next, embryo heat shock was repeated at 24 hpf and fresh 4-OHT was added. Finally, embryo heat shock was repeated at 48 hpf and fresh 4-OHT was added. Larvae were screened at 3 dpf. For heat-shock cycling, embryos were heat shocked at 8 hpf for 35 min at 37°C and transferred to a Petri dish of fresh media containing 10 μM 4-OHT for overnight incubation at 28.5°C. At 24 hpf, embryos were screened for whole body BFP expression to identify the SABER transgene. Positive embryos were transferred to individual PCR tubes containing 100 μl 10 μM 4-OHT. The embryos were heat shocked in a thermocycler for a total of 16 cycles of 25 min at 37°C and 45 min at 28.5°C. After the first four cycles, 4-OHT was refreshed, and the fish were returned to the thermocycler to complete treatment. After the last cycle (∼48 hpf), larvae were transferred to Petri dishes containing 10 μM 4-OHT and incubated at 28.5°C for 4 h, after which larvae were screened for GFP and dsRed. To test for the mortality rate associated with these conditions, wild-type TL/AB embryos were treated with either a single heat shock, heat shock cycling or no heat shock, as described above. Mortality was assessed at 2 dpf, 3 dpf, and 6 dpf with the 2 dpf measurement used as the baseline.

### Notch inhibition

*her4.3:GFP*, *her4.3:switchCas9* and *Olactb:SpCas9-GFP, 4[U6:sg]* embryos were treated with 10 µM LY-411575 or dimethyl sulfoxide (DMSO) from 4 to 8 hpf or from 4 to 72 hpf and imaged at 30, 48 and 72 hpf. After imaging, larvae were transferred to individual wells of a 24-well plate for continuous identification. Notch inhibition in the progeny of *SABER*×*her4.3:switchCas9* was performed similarly with the following modifications. At 8 hpf, embryos were heat shocked for 35 min at 37°C and transferred to a Petri dish of fresh media containing 10 μM 4-OHT and either 10 µM LY-411575 or DMSO and incubated overnight at 28.5°C. At 24 hpf, embryos were screened for whole-body BFP expression to identify the *SABER* transgene. Positive embryos were transferred to individual PCR tubes containing 100 μl of 10 μM 4-OHT and either 10 µM LY-411575 or DMSO. The embryos were heat shocked in a thermocycler for a total of 16 cycles of 25 min at 37°C and 45 min at 28.5°C. After the first four cycles, the drugs and media were refreshed, and the fish were returned to the thermocycler to continue cycling. After the last cycle (∼48 hpf), the larvae were transferred to Petri dishes containing 10 μM 4-OHT and either 10 µM LY-411575 or DMSO and incubated at 28.5°C for 4 h, after which larvae were screened for GFP and dsRed.

### Imaging

Fluorescence imaging was performed using a Leica M165 FC. Fluorescence intensity was analyzed in FIJI (Image J) 2.9.0. Corrected Fluorescence (CF) of the larval brain was measured by subtracting the product of the selection area and the mean grey value of the background from the integrated density of the selection area in each image. The Average CF (ACF) was calculated for each time point and treatment group. Confocal imaging was performed using a 40× water immersion lens on an Olympus Spinning disk confocal microscope and 3i Slidebook Software or a 20× lens on a Zeiss LSM 880 laser scanning confocal microscope. Images of adult fish were taken with a digital camera.

### Tissue dissections for gDNA analysis

*SABER*×*her4.3:switchCas9* double transgenic juvenile fish that were subject to Notch recording in early development were anesthetized and euthanized at 23 dpf using MS222 and ice. Fish were pinned on a Sylgard dish containing Neurobasal media and surgery was conducted using sterile forceps. For each fish, the brain and eyes were dissected individually. Fin and skull tissues were combined into one tube because tissue yield, especially from fins, was low. Each tissue sample was transferred into a microcentrifuge tube containing 0.5 ml 1×DPBS on ice. Tubes were briefly spun, supernatants were discarded and the tubes were flash frozen on dry ice. Nine fish were pooled per tissue sample for the juvenile timepoint. An adult double transgenic fish (∼1 year old) that was subject to Notch recording in early development was anesthetized and euthanized using MS222 and ice. The fish was pinned on a Sylgard dish and surgery was conducted using sterile forceps and scissors. The brain, eyes, fins, part of jaw and section of skin were dissected and transferred to individual microcentrifuge tubes on dry ice.

### SABER barcode amplification and gDNA sequencing

For gDNA sequencing analysis, gDNA from juvenile and adult tissues were prepared using E.Z.N.A. Tissue DNA kit according to the manufacturer's protocol. 250 ng of gDNA was used to add unique molecular identifiers (UMIs) by primer extension in 50 μl reactions using Q5 polymerase (McKenna et al., 2016). Briefly, 5 μl of 10 μM UMI primer (ACACTCTTTCCCTACACGACGCTCTTCCGATCTNNNNNNNNNNGAAACTACATGGGGTCAGCTC, where N represents random nucleotides for UMI), 250 ng gDNA, 25 μl Q5 2× Mix and water were added to 50 μl. UMI addition was carried out over two rounds as follows: 98°C for 3 min; five cycles of 66°C for 1 min and 72°C for 2 min; 98°C for 20 s; five cycles of 66°C for 1 min and 72°C for 2 min. The reaction was cleaned up using 1.5×SPRISelect beads and eluted in 30 µl elution buffer (EB). Next, SABER barcodes were PCR amplified using 1 μl of 50 μM mix of UMI_PCR F (ACACTCTTTCCCTACACGACG) and UMI_PCRR (GTGACTGGAGTTCAGACGTGTGCTCTTCCGATCTGCTGCCATTTGTCTCGAGG TC), 25 μl Q5 2× Mix and 24 μl of eluted product from the UMI extension step. The PCR reaction was as follows: 98°C for 45 s; 29 or 30 cycles of 98°C for 10 s, 67°C for 20 s and 72°C for 20 s; 72°C for 2 min. The reaction was cleaned up using 1.2×SPRISelect beads and eluted in 55 µl EB. Finally, sequencing adapters were added by PCR using 2.5 μl of 10 μM mix of UMI PCR2 P5 and P7 primers (Table S5) with 10 bp dual indices, 12.5 μl Q5 2×Mix, 2 μl eluted product from barcode PCR step and water up to 25 μl. The PCR reaction was as follows: 98°C for 45 s; 12 or 13 cycles of 98°C for 10 s, 67°C for 20 s and 72°C for 20 s; 72°C for 2 min. The reaction was cleaned up using 1×SPRISelect beads and eluted in 30 µl EB. SABER gDNA libraries were sequenced using Novaseq X+ 300 cycle kits (MedGenome) with 20% PhiX spike-in. Sequencing parameters were Read1 150 cycles, Read2 150 cycles, Index1 10 cycles and Index2 10 cycles. Standard sequencing primers were used. Sequenced reads were analyzed using the McKenna lab's SingleCellLineage package from Github (McKenna et al., 2016).

### Whole-brain scRNA-seq

Wild-type and *SABER*×*her4.3:switchCas9* zebrafish brains were harvested and dissociated at 21-23 dpf, as described previously (Raj et al., 2018b) with the following modifications. All microcentrifuge tubes were treated with 1% BSA/DPBS overnight to prevent cell loss due to adhesion. Brains were dissociated with 1 ml of 20 units/ml papain in EBSS media and incubated at 37°C for 20 min. Cells were resuspended with ∼150 μl ice-cold 1%BSA/DPBS solution. Samples were run on the 10x Chromium scRNA-seq platform according to the manufacturer's instructions (Single Cell 3′ v3 kit). Libraries were processed according to the manufacturer's instructions. Transcriptome libraries were sequenced using NovaSeq S4 300 cycle kits (Novogene) and Novaseq SP 100 and 200 cycle kits (MedGenome). Single brains were used for all experiments with whole-brain samples. In contrast, 8-10 brains were pooled for experiments where forebrain, midbrain and hindbrain regions were dissected and profiled separately.

### SABER-seq

Notch recording was performed as described above. Animals with GFP expression were raised to 21-23 dpf in a fish facility. Individual brains were dissected and dissociated as described above. To generate SABER-seq libraries, samples taken after cDNA amplification but before fragmentation were split into two parts. One part was processed for transcriptome libraries as instructed by the manufacturer. The other part was processed for SABER-seq libraries as follows. To enrich for SABER barcodes, 5 µl of the whole transcriptome cDNA was PCR amplified with 10X_p140v1UP (GTGACTGGAGTTCAGACGTGTGCTCTTCCGATCT CGTCGTAGATCTTCATCGTGATG) and 10XPCR1F (CTACACGACGCTCTTCCGATCT) primers and Q5 polymerase. The reaction conditions were: 98°C for 30 s; 14 cycles of 98°C for 10 s, 68°C for 25 s and 72°C for 15 s; 72°C for 2 min. The reaction was cleaned up with 0.8× SPRISelect beads and eluted in 20 µl EB. A second PCR was carried out using 3 µl of PCR1 product, and 10XPCR1F and 10X_p140v2Sa (GTGACTGGAGTTCAGACGTGTGCTCTTCCGATCTGAAACTACATGGGGTcaGCTC) primers. Reaction conditions were the same as PCR1 except only 13 cycles were completed. At this point, two different clean up conditions were used: iteration 1 (SABER-seq_v0) and iteration 2 (SABER-seq_v1). For iteration 1, the reaction was cleaned up with 0.8× SPRISelect beads and eluted in 20 µl EB similar to PCR1. For iteration 2, an optimization was trialed, and the reaction was cleaned up with right side size selection using SPRIselect beads (0.6×beads used initially and supernatant was rebound with 1.5×beads). The product was eluted in 20 µl EB. Finally, adapters and sample indices were incorporated in a third PCR reaction using 5-7 µl of a 1:10 dilution of PCR2 product. Reaction conditions were the same as PCR1, except nine or ten cycles were completed. Primers used were: 10XP5Dual_Hx (AATGATACGGCGACCACCGAGATCTACAC-xxxxxxxxxx-ACACTCTTTCCCTACACGACGCTCTTCCGATCT), 10XP7Dual_H5 (AATGATACG GCGACCACCGAGATCTACAC-xxxxxxxxxx-ACACTCTTTCCCTACACGACGCTCTTCCGATCT) where x represents index bases from the 10X Dual Index Kit TT, SetA H index entries (Table S5). For iteration 1, the third PCR reaction was cleaned up with 0.8×SPRISelect beads and eluted in 20 µl EB to generate final libraries. For iteration 2, the third PCR reaction was cleaned up with right side size selection using SPRIselect beads (0.65×beads used initially and supernatant was rebound with 1.5×beads). SABER-seq libraries were sequenced using Novaseq SP 200 cycle kits (MedGenome) with 5-10% PhiX spike-in. Sequencing parameters: Read1 28 cycles, Read2 151 cycles, Index1 10 cycles and Index2 10 cycles. Standard sequencing primers were used.

### Transcriptomic data analysis

Raw FASTQ reads were aligned against zebrafish genome, built from GRCz.109.gtf and the GRCz11.fa files, using the Cell Ranger count pipeline to generate gene-by-cell count matrices (filtered barcode matrix). The count matrices were processed using the standard workflow of the Seurat (v5.0.2) R package with modifications. Briefly, all count matrices were separately loaded into R. Seurat objects were then created with modified parameters, requiring features to be detected in at least three cells and cells to contain at least 300 genes. Subsequently, the Seurat objects were merged to create a single Seurat object. Cells with RNA counts between 300 and 4000, and a mitochondrial gene percentage of less than 9% were selected for downstream analysis. Gene expression values were log-normalized with the default parameters and scaled for the top 3000 variable genes. The scaled data were then used to compute the 50 principal components. Cells were iteratively clustered using the Louvain algorithm after identifying neighboring cells. Differentially expressed genes for each cluster were identified using genes that are detected in at least 10% of cells in either of the two clusters and genes showing a minimum difference of 10% between the two clusters. Cell types were annotated using known marker genes. Finally, the data were visualized using UMAP.

### SABER-seq barcode analysis

The SABER-seq FASTQ reads were processed using the Cell Ranger count pipeline, similar to the transcriptomic reads. A bash script was then used to generate a text file containing all FASTQ headers and error-corrected cell barcodes for SABER-seq data. After loading filtered barcode matrices into R, error-corrected cell barcodes from transcriptomic reads were isolated and matched against those in the text file to identify common cell barcodes that are shared between the transcriptomic and SABER-seq datasets. The resulting text file contained redundant error-corrected cell barcodes associated with different UMIs for the same cell. Thus, unique error-corrected cell barcodes were isolated to create a text file containing unique FASTQ headers and error-corrected barcodes. Subsequently, FASTQ reads matching the FASTQ headers in this file were extracted from the SABER-seq raw reads using a Python script. SABER-seq barcodes were analyzed using the scGESTALT pipeline available on Github as described previously (Raj et al., 2018a, 2020).

### qPCR

TL/AB embryos were treated with 10 µM LY-411575 or DMSO from 4 to 72 hpf and fixed at 72 hpf. Twenty embryos were pooled per condition. RNA was extracted using Trizol according to the manufacturer's guidelines. cDNA was prepared from 1 µg of RNA using RevertAid RT Reverse Transcription Kit according to the manufacturer's guidelines. qPCR reactions were performed using Power SYBR Green PCR Master Mix and a Quant Studio 3.0 machine. CT values corresponding to transcript abundance were normalized to the *beta-actin* reference gene and the fold change was calculated by 2^(−ΔΔCT). Data from three biological replicates were used for analyses.

### HCR RNA-FISH

HCR probes were designed according to the Ozpolat lab's instructions available on Github: https://github.com/rwnull/insitu_probe_generator. The probe for *elavl3* was obtained from Molecular Instruments and probes for *aldoca* and *neurod1* were ordered as 50 pmol pools from IDT. HCR buffers and hairpins were procured from Molecular Instruments. The experiments were performed as previously described (Choi et al., 2018), along with modifications described by Molecular Instruments. Briefly, DMSO control and Notch inhibited samples were fixed overnight with 4% paraformaldehyde at 72 hpf at 4°C with gentle shaking. Samples were then dehydrated with 25%, 50%, 75% and 100% methanol, and stored in methanol at −20°C until used. Samples were processed with PBST, proteinase K digestion and PFA fixation followed by prehybridization with hybridization buffer at 37°C in a thermomixer with gentle agitation at 300 rpm for 30 min to 2 h. The samples were then hybridized with probes for *aldoca*, *neurod1* and *elavl3* at a final concentration of 1 µM at 37°C in a thermomixer at 300 rpm for 12-16 h. The samples were washed with probe wash buffer and 5×SSCT and pre-amplified with amplification buffer in a thermomixer at 24°C for 30 min to 2 h followed by amplification with snap cooled hairpins (3 µM working concentration) for 12-16 h in a thermomixer set at 24°C and 300 rpm. Samples were washed with 5×SSCT and PBST, and then stained with DAPI followed by subsequent PBST washes. Imaging was performed using the Zeiss Laser confocal microscope LSM880 and Zen Black software with appropriate laser powers for 405 nm, 488 nm and 561 nm at 10× magnifications. Images were processed with Fiji ImageJ software. Probes are available in Table S5.

### Statistical analysis

Statistical analysis was carried out in R Studio Build 402. To analyze differences in fluorescence intensities in Notch-inhibited groups, the Mann–Whitney *U*-test was used. To analyze differences in mortality during heat shock treatments, one-way ANOVA was used. To analyze differences in SABER barcode detection and editing frequencies between cell types, the binomial test was used. To analyze differences in gene expression via RT-qPCR, an unpaired two-tailed *t*-test was used.

## Supplementary Material



10.1242/develop.203102_sup1 Supplementary information

Table S1. List of edited and unedited SABER-seq barcodes recovered from three zebrafish brains.

Table S2. List of marker genes from scRNA-seq clustering analysis of 21-23 dpf zebrafish brains.

Table S3. List of references for scRNA-seq cluster identification.

Table S4. List of edited and unedited SABER-seq barcodes matched to corresponding cell types and list of cell types with bias in barcode detection.

Table S5. List of oligos, probes and SABER barcode sequence.
