# Supplementary material for: Barcoding Notch signaling in the developing brain
Source: Development. 2024 Dec 20;151(24):dev203102. doi: 10.1242/dev.203102 (PMC11701514; doi:10.1242/dev.203102)
Supplement: Supplementary information [file develop-151-203102-s1.pdf]

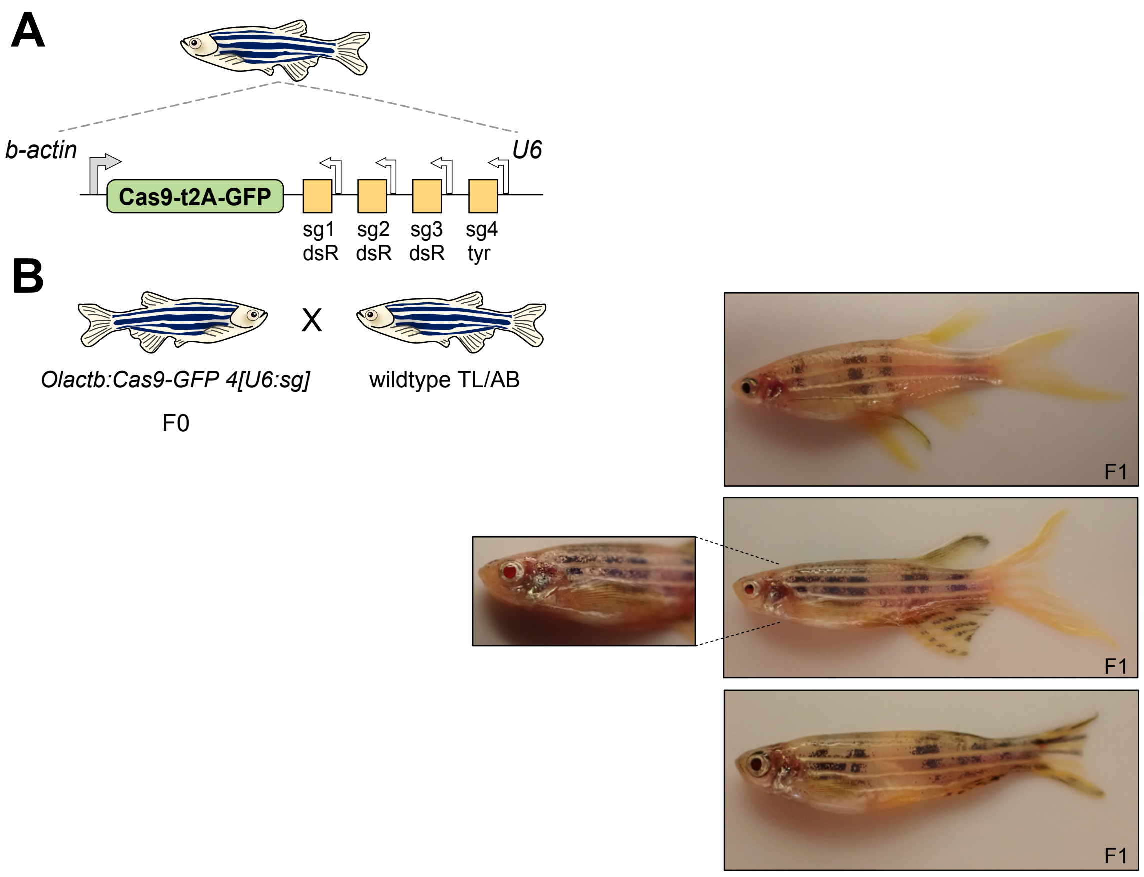

Fig. S1. *Olactb:Cas9-GFP, 4[U6:sg]* transgenic adults have extensive defective pigmentation.
A) Schematic of *Olactb:Cas9-GFP, 4[U6:sg]* transgenic reporter. sg, sgRNA. sg4 targets *tyr* gene. sg1-3 target dsRed sequence.
B) Left, schematic of *Olactb:Cas9-GFP, 4[U6:sg]* outcross. Right, whole-body images of adult zebrafish with defective pigmentation in F1 adults. Dotted lines represent zoomed-in image of disruption of eye pigmentation. These transgenic animals have more extensive pigment defects compared to *tp1:Cas9-GFP, 4[U6:sg]* animals due to ubiquitous expression of the *b-actin* promoter.

Development • Supplementary information

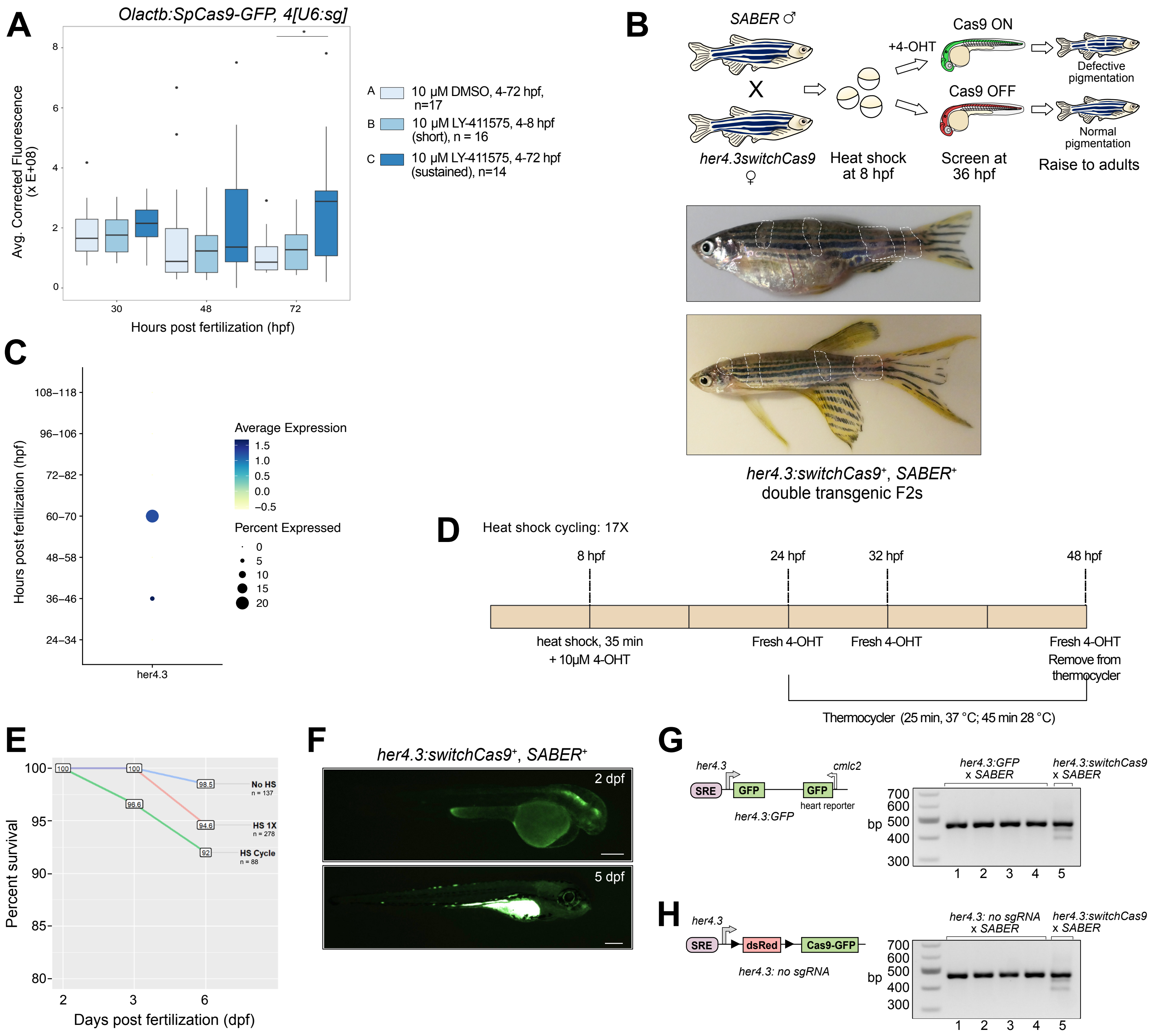

Fig.S2. SABER, a novel CRISPR-Cas9 signal recorder.

A) GFP fluorescence intensity in *Olactb:Cas9-GFP, 4[U6:sg]* embryos after Notch inhibition. Embryos were treated with DMSO control at 4-72 hpf, LY-411575 inhibitor at 4-8 hpf (short pulse), and LY-411575 inhibitor at 4-72 hpf (sustained). Images were taken at 30, 48 and 72 hpf. n, number of embryos. *, $p < 0.05$, Mann-Whitney U test.

B) Whole-body images of double transgenic F2 adult zebrafish from *her4.3:switchCas9* x *SABER* cross. Embryos were treated with heat shock and 4-OHT to activate CreERT2 and turn on Cas9 expression in a Notch and *her4.3* dependent manner (top panel). Only GFP positive fish developed minor pigmentation defects. Dotted lines indicate areas with disrupted pigmentation in stripes.

C) Dot plot of *her4.3* gene expression in xanthophore cells across various developmental stages. Data is from Sur et al. 2023, *Developmental Cell*.

D) Schematic of heat shock cycling conditions.

E) Survival rates of TL/AB fish after heat shock treatments. HS, heat shock. No HS, control. HS 1X, heat shock once at 8 hpf. HS cycle, heat shock cycling as shown in D). No statistical difference, p = 0.443, one-way ANOVA test.

F) Fluorescence images of *her4.3:switchCas9⁺; SABER⁺* larvae at 2 and 5 dpf. Scale bar, 250 µm.

Genomic DNA amplification of SABER barcodes at 3 dpf to assess barcode editing in the absence of (G) Cas9 and sgRNA cassette and (H) sgRNA cassette. Each lane represents one larval fish.

Development • Supplementary information

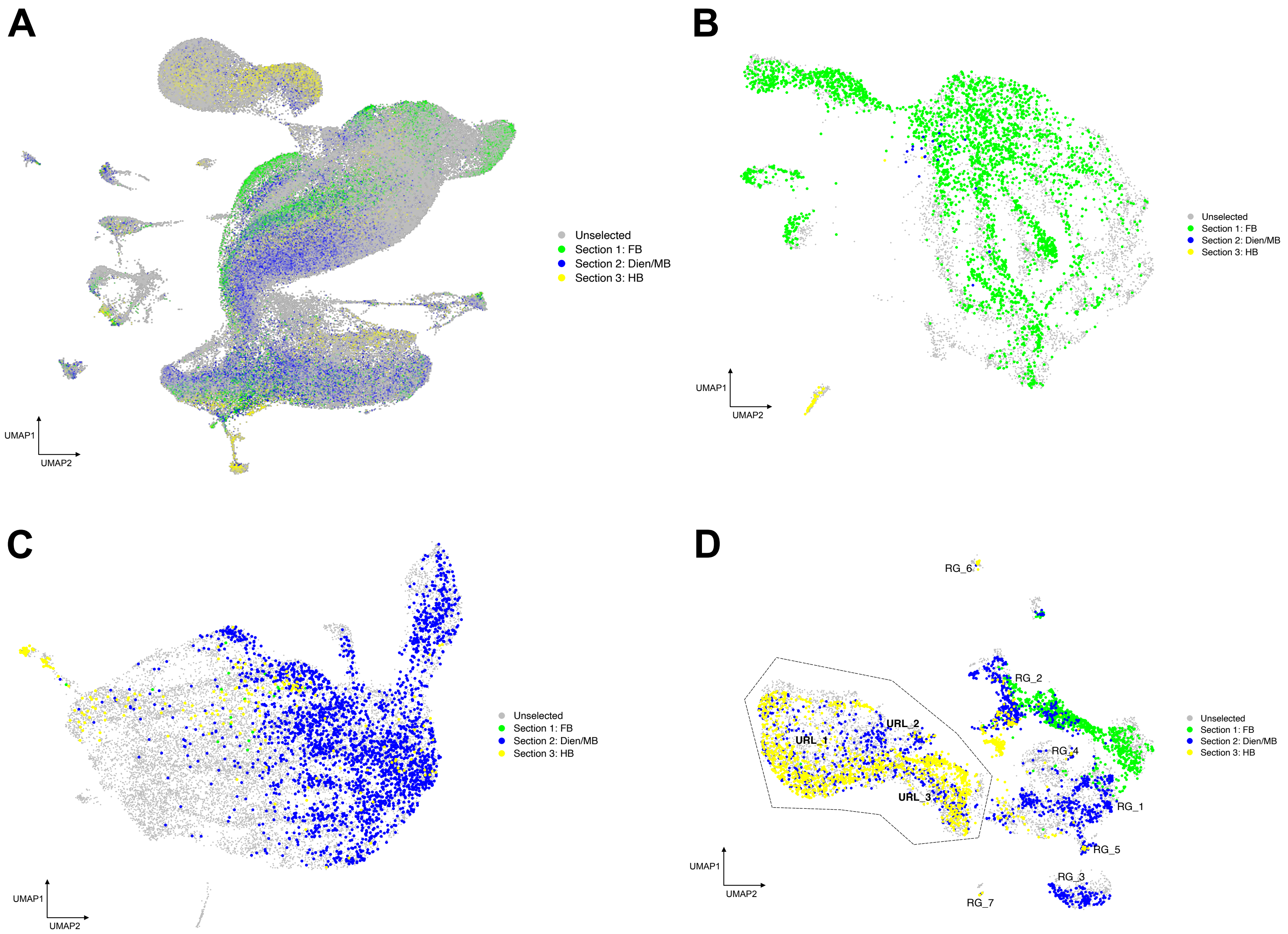

Fig. S3. Overlap of brain clusters with dissected regions.

A) UMAP embedding of 148,853 cells with cells from corresponding dissected regions highlighted. Brains from multiple fish were manually dissected into 3 coarse regions: forebrain (FB), diencephalon + midbrain (Dien/MB), hindbrain.
B) Overlap of pallium subclusters with forebrain dissected cells.
C) Overlap of midbrain subclusters with diencephalon/midbrain dissected cells.
D) Overlap of upper rhombic lip subclusters (dotted outline) with hindbrain dissected cells.

Development • Supplementary information

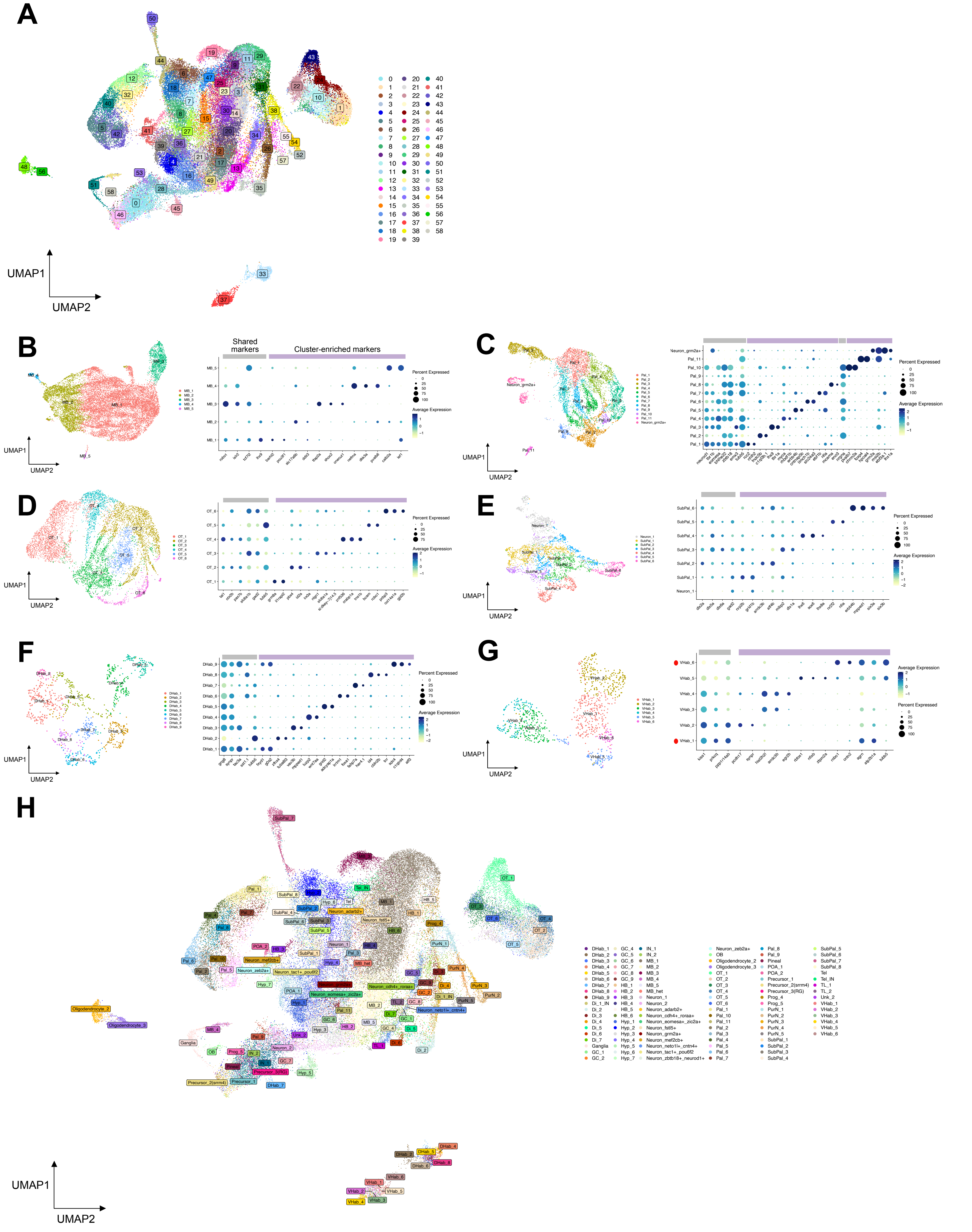
A
UMAP1
UMAP2
B
Shared markers
Cluster-enriched markers
Percent Expressed
Average Expression
C
D
E
F
G
H

Fig. S4. Brain cell type classification.

A) UMAP embedding of 59 subclusters obtained after additional subclustering analysis of select neuron and oligodendrocyte clusters identified from the initial coarse-grained clustering shown in Fig. 5A.
UMAP embedding and dot plots of marker gene expression from subclustering of B) midbrain cells, C) pallium cells, D) optic tectum cells, E) subpallium cells, F) dorsal habenula cells, and G) ventral habenula cells. Red dots represent an immature cell state (vHab_6, high tubb5 and low kiss1 expression) and a corresponding mature cell type (vHab_1, low tubb5 and high kiss1 expression).
H) UMAP embedding as described in A) with subcluster identities overlaid.

Development • Supplementary information

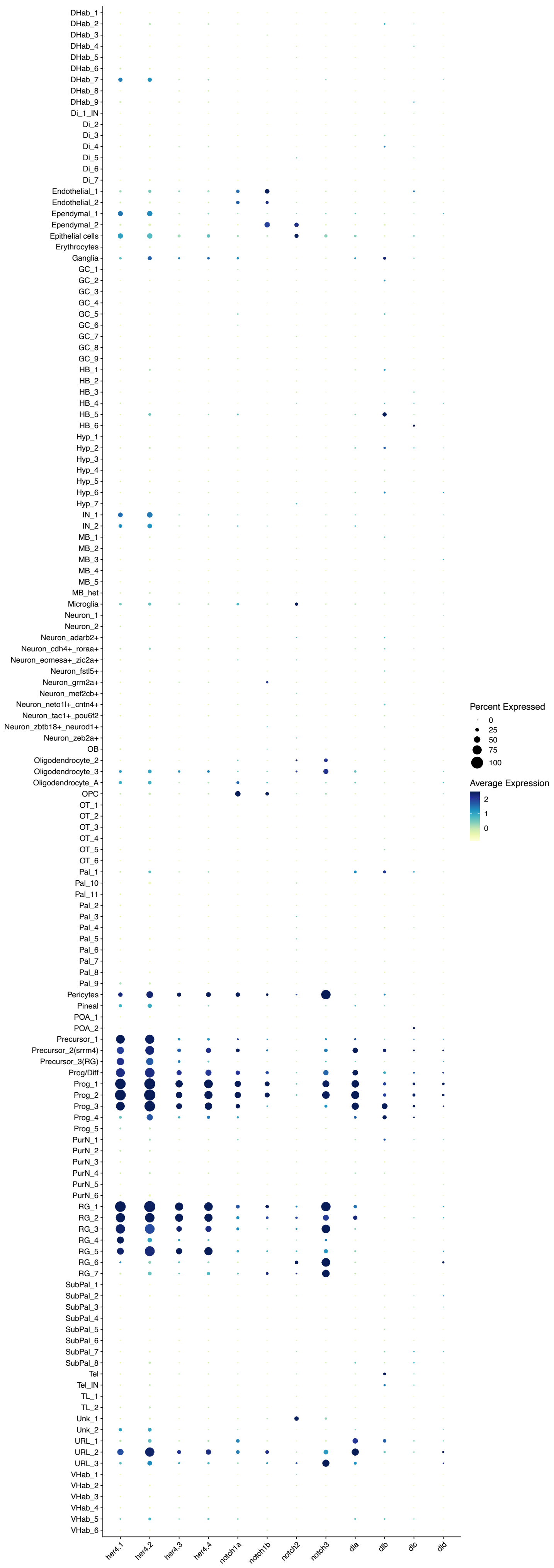

Fig. S5. Gene expression dot plot of *her4* variants, *notch* genes and *delta* genes across all subclusters.

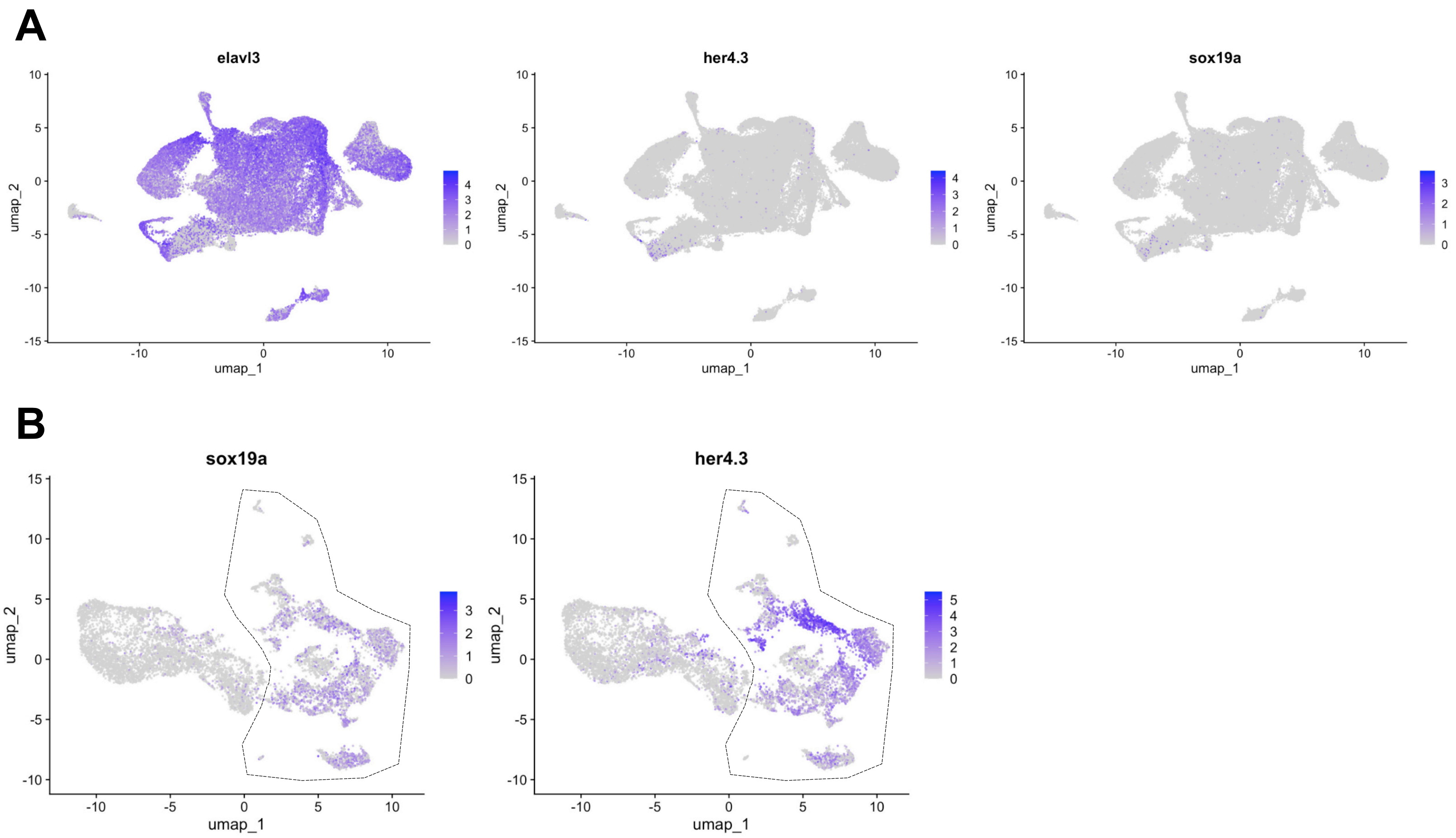

Fig. S6. Marker gene expression patterns in brain scRNA-seq dataset.

A) UMAP embedding of neuronal and oligodendrocyte subclusters (see also Fig. S4A, S4H) showing expression of neuronal marker *elavl3* and neural progenitor markers *sox19a* and *her4.3*.

B) UMAP embedding of radial glia and upper rhombic lip progenitor subclusters (see also Fig. 5D) showing expression of *sox19a* and *her4.3*. Radial glia subclusters are indicated by a dotted outline.

Development • Supplementary information

Table S1. List of edited and unedited SABER-seq barcodes recovered from three zebrafish brains.

Available for download at
https://journals.biologists.com/dev/article-lookup/doi/10.1242/dev.203102#supplementary-data

Table S2. List of marker genes from scRNA-seq clustering analysis of 21-23 dpf zebrafish brains.

Available for download at
https://journals.biologists.com/dev/article-lookup/doi/10.1242/dev.203102#supplementary-data

Table S3. List of references for scRNA-seq cluster identification.

Available for download at
https://journals.biologists.com/dev/article-lookup/doi/10.1242/dev.203102#supplementary-data

Table S4. List of edited and unedited SABER-seq barcodes matched to corresponding cell types and list of cell types with bias in barcode detection.

Available for download at
https://journals.biologists.com/dev/article-lookup/doi/10.1242/dev.203102#supplementary-data

Table S5. List of oligos, probes and SABER barcode sequence.

Available for download at
https://journals.biologists.com/dev/article-lookup/doi/10.1242/dev.203102#supplementary-data
