# Supplementary material for: Barcoding Notch signaling in the developing brain
Source: Development. 2024 Dec 20;151(24):dev203102. doi: 10.1242/dev.203102 (PMC11701514; doi:10.1242/dev.203102)
Supplement: Supplementary file 1 [file develop-151-203102_review_history.pdf]

Barcoding Notch signaling in the developing brain

Abigail M Siniscalco, Roshan Priyarangana Perera, Jessie E Greenslade, Hemagowri Veeravenkatasubramanian, Aiden Masters, Hannah M Doll and Bushra Raj
DOI: 10.1242/dev.203102

Editor: Debra Silver

Review timeline

Original submission:	29 May 2024
Editorial decision:	8 August 2024
First revision received:	21 October 2024
Accepted:	10 November 2024

Original submission

First decision letter

MS ID#: DEVELOP/2024/203102

MS TITLE: Barcoding Notch signaling in the developing brain

AUTHORS: Bushra Raj, Abigail M Siniscalco, Roshan Priyarangana Perera, Jessie E Greenslade, Aiden Masters, and Hannah M Doll

I have now received all the referees' reports on the above manuscript, and have reached a decision. My apologies for the delay in getting these reviews to you - we ran into significant challenges with finding reviewers. The referees' comments are appended below, or you can access them online: please go to BenchPress and click on the 'Manuscripts with Decisions' queue in the Author Area.

As you will see, the referees express considerable interest in your work, but have some significant criticisms and recommend a substantial revision of your manuscript before we can consider publication. Reviewers 1 and 2 raise significant concerns about the widespread utility of this approach. It will be essential to address this not only with some text but also with additional new analyses/validations. Reviewer 1 asks for analysis of a second tissue for proof of principle and reviewer 3 also asks for critical biological validation experiments. Addressing these (and other) concerns raised by the reviewers will be necessary to support the value of your study for the Development community. Reviewer 2 raises points that will be valuable to clarify and address as feasible.

If you are able to revise the manuscript along the lines suggested, which may involve further experiments, I will be happy receive a revised version of the manuscript. Your revised paper will be re-reviewed by one or more of the original referees, and acceptance of your manuscript will depend on your addressing satisfactorily the reviewers' major concerns. Please also note that Development will normally permit only one round of major revision. If it would be helpful, you are welcome to contact us to discuss your revision in greater detail. Please send us a point-by-point response indicating your plans for addressing the refereesâ€™ comments, and we will look over this and provide further guidance.

Please attend to all of the reviewers' comments and ensure that you clearly highlight all changes made in the revised manuscript. Please avoid using 'Tracked changes' in Word files as these are lost in PDF conversion. I should be grateful if you would also provide a point-by-point response detailing

© 2024. Published by The Company of Biologists under the terms of the Creative Commons Attribution License (https://creativecommons.org/licenses/by/4.0/).

how you have dealt with the points raised by the reviewers in the 'Response to Reviewers' box. If you do not agree with any of their criticisms or suggestions please explain clearly why this is so.

Reviewer 1

Advance summary and potential significance to field

In their manuscript "Barcoding Notch signaling in the developing brain", Siniscalco et al. present SABER-seq a CRISPR/Cas9 based molecular recorder for storing developmental signaling cues as permanent mutations in cellular genomes for later readout by scRNA-seq. They apply SABER-seq to record Notch signaling in developing zebrafish brains. Molecular recording is an exciting emerging technology, and this manuscript constitutes an important step towards establishing powerful recording systems. However, the performance of the system presented here, while clearly going beyond the published state of the art, is still affected by considerable limitations regarding the dynamic range (binary readout), the temporal control (long induction needed), and the biological interpretation (see major points 1 and 2). While these limitations certainly restrict the biological application range, I would consider this manuscript to be suitable for publication in Development, since it is not only one of the first manuscripts to explore molecular recording of signaling cues, but it also includes careful characterization and calibration of the system. The following comments may help improve the manuscript further.

Comments for the author

Major comments:

1) An interesting application of such a recording system would be to relate signal exposure in specific time windows to the resulting cell fate decisions. This question is only addressed at the very end of the manuscript, in Fig. 5d. This part should be expanded. In particular, as presented, it is not clear how big the differences between different cell types are in terms of the fraction of edited barcodes.

2) It looks as if the fraction of edited barcodes does not vary much between the different cell types of the brain. Since one main focus of this manuscript is to introduce a novel methodology, it would be important to also analyze another tissue (or another period of induction, or another stage of the fish) in which there is a clearer relationship between signal exposure and cell type. This would be required for a proof of principle.

3) In Fig. 2i, the authors demonstrate that heatshock cycling is necessary to achieve high levels of editing. Does this rather harsh treatment have any negative effects, e.g. developmental defects or increased mortality? Can you pleases describe how you tested for adverse effects or how you prevented them from occurring?

Also, can you please specify if the heatshock cycling was performed in the same way (and for the same duration) in the 3 dpf and 3 wpf samples? Observing strong editing after the end of induction would be a more direct argument for ongoing recording than continuous detection of GFP (as discussed in line 197).

Minor comments:

4) It would be good to state explicitly already at an earlier stage in the manuscript that the dynamic range is limited and that you go for a binary analysis. Otherwise the reader might worry about the tandem repeats in the SRE (non-linearity), Cas9 excisions of previously recorded events, or other features that could reduce resolution, but are not of major importance for the current binary readout.

5) Fig. 5b: Maybe you can consider highlighting not only the cells with detected SABER barcode, but also highlight those with an edited barcode in a different color. Right now, the statement in line 317 ("Accordingly, we detect edited SABER barcodes in neural progenitors, immature neurons and mature neurons (Fig. 5b)") is not really apparent in the figure.

© 2024. Published by The Company of Biologists under the terms of the Creative Commons Attribution License (https://creativecommons.org/licenses/by/4.0/).

Also, I found the statement in the figure caption that cells with barcodes are shown in red confusing, since not all of these dots appeared in red in the pdf (this might be a result of figure compression).

6) Guide RNA targets: Could the authors please clarify the targets of the guide RNAs in the experiments described in figure 1? In lines 118-120 the authors mention inclusion of a guide RNA against tyr – is this guide RNA one of the ones shown in figure 1b? Additionally, is the guide RNA against tyr retained in the her4.3:switchCas9 fish, resulting in a total of five guide RNAs in those fish?

7) Lines 75-78: “In addition to lineage analyses, studies have applied molecular recorders to record a wide array of cellular and synthetic events such as gene expression, cellular stimuli, synthetic signals, videos, and text messages but these have been limited to cell culture systems or prokaryotic cells and have not been combined with scRNA-seq” - This sentence describes very different applications followed by eight sources. Maybe it’s worth separating biological and technical applications to improve clarity?

8) Lines 302-304: Is there a specific reason the authors chose beta-actin to drive the recorder transgene?

9) Figure 3c: Are cells where the entire target is deleted included under the intersite category? Targets 2 and 3 seem to have been excised in some cases according to fig. 3d.

Reviewer 2

Advance summary and potential significance to field

In this manuscript, Siniscalco and colleagues present the Â“SABER-seqÂ” system to record signalling history during embryo development. In short, Cas9 expression is triggered by the signal of interest, here Notch, and causes mutations in a barcode. Subsequently, single-cell sequencing is applied on the embryo to concurrently map cell types and detect edited barcodes, which indicate that Notch signalling occurred at some point in that cell or its progenitors.

Comments for the author

I very much enjoyed reading this manuscript. It demonstrates a creative and original solution to recording signalling activity during development. I found this work inspiring and I am confident other readers will too. The presentation and writing style are of high quality. This makes the paper more digestible, which is much appreciated as some of the genetics is not trivial.
In my opinion, the paper could be published in Development with no edit. Here are some suggestions for the authors if they wish to further improve the paper.

--- On the science ---
– A potential pitfall of the approach is DNA repair not being equal in different cell types or across development or at different stages of cell cycle. Namely, I think the approach somewhat assumes that double-strand breaks (DSBs) are always repaired in the same way, but this is unlikely to be the case. While perhaps not a crucial consideration now, it would become important if making the approach more quantitative in the future, as suggested in Discussion. When Cas9 is injected at the one-cell stage, embryos mainly rely on alternative end-joining to repair DSBs (cf. Thyme 2016), but I doubt this remains the case for long as it is highly error-prone. Non-homologous end-joining, for example, is more accurate, i.e. leaves fewer indels. Accordingly, I think the sensitivity of SABER would (annoyingly) probably vary between different cell types or developmental stages?
As in, a little pulse of Notch signalling in a cell that uses non-homologous end-joining may leave no trace in the barcode, while the same pulse may leave indels in a cell that uses alternative end-joining. This point could be elaborated upon in Discussion, maybe around line 399.

– Silencing of Cas9 expression is a limiting factor, not only for this approach.
Would insertion of the transgene in one of those pIGLET safe harbor loci help in any ways?

© 2024. Published by The Company of Biologists under the terms of the Creative Commons Attribution License (https://creativecommons.org/licenses/by/4.0/).

- About making the system more quantitative, maybe it is possible to find a hint of this possibility in the current data? My logic would be: one could probably estimate the number of double-strand breaks for each “barcode allele”, giving a signal from 0 to 4. Now, if we can identify a few cell types which are known to have received subdued/brief Notch signalling, do they tend to have lower number of double-strand breaks? What about cell types with continuously high Notch signalling, do they all have alleles which are consistent with 4 double-strand breaks (e.g. small indel at each site)? Maybe some version of this idea is the way to go for quantitative recording? You would probably want to prevent large deletions somehow (as e.g. a large deletion between target 1 and 4 makes it impossible to count the ‘real’ number of double-strand breaks, anywhere from 2 to 4), maybe with that DNTT polymerase or inhibition of alternative end-joining (see inhibitor ART-558, but ideal would be fusing some inhibitor to the Cas9 from the transgene).

- To record multiple signals, would it not be possible (and much easier/scalable than expressing different Cas proteins) to simply express different gRNAs and have a bigger barcode with more targets? Maybe there is a simple reason why this does not work…

- There are many steps before the first indels occur, in chronological order: Cre transcription + translation, Lox-P excision, Cas9 transcription + translation assembly with sgRNA, double-strand break, repair (probably multiple cycles of break & repair as the repairs may be correct most of the times). Any guess on how long it all takes? This would impact experimental designs/interpretation of results, e.g. it might be that there is a 6-hour lag between start of recording and first indels. I guess you could fairly easily test this by starting recording then extracting genomic DNA at successive timepoints. How early can you detect indels?

- Results in Fig. S2: I am not sure I understood the pigmentation defects in those fish. In my understanding, the full SABER system does not have a gRNA targeting tyr anymore, as that was just to test the system and we do not want to create mutations in endogenous sequences?

- I was slightly confused by the transition to the section starting at line 306.
Do I understand correctly that 126/127 of cell clusters had edited barcodes? It sounds a bit odd to check whether they are the “expected” cell types if that is (virtually) all of them and there is no negative control, so to speak. What is this one cluster that did not have edited barcodes? Maybe that is interesting?

--- On the writing etc. ---
- Fig. 2i: I would not label the two bands “edited” and “unedited”. The majority of edits may well be small indels (this is certainly the case when injecting one gRNA), which would not be easily detected on the gel.

- line 62, I see this is common usage, but I am slightly confused by “single-cell genomics”. I think we are mainly referring to transcriptomics here? I would understand single-cell genomics as sequencing genomes of single cells. I think “single-cell sequencing or transcriptomics” would be better.

- I think the writing around 168–173 could be a bit more explicit to help readers.
Something like: “to trigger … excision of the dsRed and stop codon, allowing Cas9 expression”; then “GFP expression in the brain, indicating successful excision of the dsRed and Cas9 expression”.

- Around 184–188, I would use the transgene names introduced before? e.g. “single transgenic” is SABER, right?

- Line 205: “switching on Cas9-GFP expression when Notch activity is inhibited”, I was slightly confused by this wording. In theory, Cas9-GFP expression should be off when Notch is inhibited. How about “switching on recording when…”?

- Results & Figure legend: stripe “mutations”, I would say “defects” or else? To keep mutations as meaning DNA mutations.

Reviewer 3

© 2024. Published by The Company of Biologists under the terms of the Creative Commons Attribution License (https://creativecommons.org/licenses/by/4.0/).

Advance summary and potential significance to field

This work presents a new method to record developmental signals. A notch-responsive CRISPR/Cas9 makes edits to a barcode (in this case 4 sgRNA sites), and the edits are sequenced alongside single-cell RNA-sequencing to determine whether the cell experienced notch signaling. In addition to this tool, a new atlas of the 21-23 dpf zebrafish brain (almost 150k cells) was included. This is a well-written manuscript with clear figures and presentation. Conceptually, the tool is interesting, and I commend the authors on making progress on this interesting technical challenge. There are, however, some aspects of the tool and conclusions that need further validation. Given the technical issues and questions that remain, it is unclear whether it would be adopted by the field when other options exist. The biological demonstration of the tool, more or less editing in cell types that receive differential notch, is also not sufficiently validated. Finally, while the new scRNA-seq data is itself extremely valuable to the community, there are issues with its annotations and the depth of analysis completed. Building a new atlas is obviously not the goal of this work, but publishing inaccurate annotations could lead others in the field astray.

Comments for the author

Major comments
While I understand there is novelty in using CRISPR, as well as the possibility to scale the concept with orthogonal systems, the method still feels somewhat preliminary. One main issue is that only seems able to measure the on/off state of signaling, or at least there was no attempt to determine whether edits to multiple sites in the barcodes had any meaning as far as signaling level. The concept presented is that the number of cells in a cluster that are edited is a proxy of signaling for that cluster. That is possible, but it would be much better to be able to have some idea of signaling level. This is a first pass at a challenging problem, so the method is still worth considering despite this point. There are also other challenges that came up, though, with only up to 20% of the barcode sequences even being recovered using this method. Given the experimental challenge of needing to recover the DNA barcode separately from the RNA sequencing, and that it is not working very well, I wonder if this approach is really a good way to accomplish the research goal. Since an on/off signal is all that is desired, perhaps measuring the RNA from a very stable and highly-expressing promoter would be better. For example, making Cre notch-responsive instead of Cas9, and then flipping on expression of a stabilized RNA that is under control of b-actin, could be an alternative that might be less technically challenging and give better recovery of the signal. With the current success of the method, I am left wondering why this is a good idea compared to alternatives.

My biggest concern is regarding whether the edits observed in the sequencing are really from notch-responsive events. The her4.3 promoter is supposed to be responding to notch, particularly in the development of the brain. The edits appear to be equally distributed, which is mentioned by the authors, and even appear to be found in blood cells. There are also pigment disruptions in the her4.3 promoter fish, which makes sense given the presence of edits in every cluster. The supposition that these edits are due to notch signaling rests heavily on the single gel in Figure 2i. Additional information added to that gel would be helpful: what do the editing profiles look like in non-neuronal tissues at 3 wpf and what does the control look like at 3 wpf? The gel is clean and fairly convincing, but the presence of these edits throughout so many non-neuronal cell types is not consistent with what we know about her4.3 expression.
It is possible that it would be transiently expressed in those types, but then I think that one would expect a lower level of editing compared to types with much more sustained expression. The discussion states that “her4.3:switchCas9 was expressed in ancestors of melanophores”. There are ways to check this point, as well as the other non-neuronal cell types that display unexpected editing, such as imaging of their existing switch reporter line in combination with HCR to label cell types. Even if the GFP signal degrades, there should still be a sufficient time window to determine whether the lox sites are being edited outside of the brain as well.

The point about identifying possibly different notch responsiveness in the TL/GC subtype compared to the Purkinje type is interesting. It is, however, not very convincing in light of the previous point about the edits being everywhere regardless of how likely it is for her4.3/notch to be involved in their development. In the condition of notch inhibition over the identical time window used for

© 2024. Published by The Company of Biologists under the terms of the Creative Commons Attribution License (https://creativecommons.org/licenses/by/4.0/).

these editing experiments, are certain cell types more or less likely to be impacted? An HCR following inhibition could give insight into this point.
Also, on a related point, approximately half of the barcodes were recovered from each of two experiments. Were there any differences in which cell types had barcodes in each experiment (such as this 5D finding, did it replicate in both experiments)? It is probably possible to come up with a biological system where it is known that notch is not required in some cells and not in others, and that may be an option for a biological demonstration of the tool's effectiveness.

I have significant concerns with some of the cell type assignments. For example otpa/otpb are mainly markers of the peoptic area, not the subpallium (line 267).
It is possible that further subclustering could resolve some of the issues.
Other genes in this cluster (e.g., bsx) also indicate that it is preoptic area or that it requires further subclustering. Another example that is a little confusing is the galn in the TL. While I do see some potential literature on this, it is not reported in the zebrafish brain and larval in situ is not compatible with this idea. Third, the cluster labeled Hypothalamus 5 with high expression of synpr and sp8a lines up with a known subpallium cluster. What is the evidence that this cluster is from the hypothalamus? Fourth, Pallium_9 is strongly labeled by grm2a, which is an olfactory bulb marker gene. In summary, I would suggest making a detailed supplementary table with references to support each of the cluster annotations to support the point on line 259 that markers from the literature were used to assign types. Additionally, to support the point that previous datasets (references 36-38) were used for subregions, I suggest actually showing an explicit comparison of the clusters such as with a random forest plot. Additionally, there are also large brain regions not listed in the analysis, such as the tuberculum, thalamus, and hindbrain other than the granule cells (there are existing scRNA-seq datasets for this to compare to for this area). I suggest carefully going through the brain and cell type annotations to ensure their accuracy. I did not go through every cluster and proposed cell type, but I expect that there are more that either need justification or correction.

Related to the comment above. Both dissected and whole brain data are included in the new scRNA-seq dataset presented here. However, no analysis was done to show the overlay of the dissected areas on those cell types, as far as I can tell. It would be very helpful to be able to match up the brain regions with the cell types, to further support their classifications. I understand that this is not the main point of the manuscript, but accurate annotations are important for any published dataset.

Minor comments
Line 37: I am not sure I agree with the word “accumulates” in the abstract given the emphasis later on about how this is an on/off label (edited/not edited). I think accumulates implies that the mutation count would increase with signal, which is not necessarily shown in this work.

Lines 56-49: Why are imaging-based readouts limited to a predefined area? The entire brain can easily be imaged in zebrafish.

Line 163 (and possibly more): Check for the use of apostrophe versus the primer symbol.

Line 199: Why is this data not shown? It seems like a useful point. I generally don't agree with including statements about findings if there is no data to support them.

Line 221 and throughout: 10X is not correct (not large X).

Line 242/243: Seems not necessary in the Results section. The following sentence works fine as a topic sentence for that paragraph.

Figure 2: Why is Figure S2D in the supplement instead of along with 2i? It seems like a very important point (main text worthy) that the drug molecule reduces editing, showing that the editing is notch responsive.

Line 499: The plasmids are not currently on addgene, and the sequences are not provided as part of the manuscript supplementary information. While it mostly does not matter, the spacing between

© 2024. Published by The Company of Biologists under the terms of the Creative Commons Attribution License (https://creativecommons.org/licenses/by/4.0/).

the guide RNAs and their sequences are not provided. This information would have been helpful for the review, such as for interpretation of the sequences in Table S1.

Figure 4A: The colors do not appear to line up with the labels. Epithelial cells is supposed to be brown and pericytes are missing.

The tab in Table S2 labeled PurN is empty, and the PurN neurons are in the Progenitors tab.

Table S2: What does a cluster label of “removed” mean. Why was this cluster removed?

Figure S3A: hypothalamus is misspelled. Why are the hypothalamus and diencephalon separately labeled? The diencephalon contains the hypothalamus.
Also, why are some labeled and not others. There doesn’t appear to be a lot of reason for what is labeled and not, and it’s hard to tell what the cluster the label is referring to.

Check the entire manuscript for appropriate italics on gene names.

I was not given a reviewer token for the data on GEO. While this may not be the fault of the authors, it will be critical to ensure that all data is available on GEO and clearly marked before publication.

First revision

Author response to reviewers' comments

Reviewer 1 Advance Summary and Potential Significance to Field:

In their manuscript "Barcoding Notch signaling in the developing brain", Siniscalco et al. present SABER-seq, a CRISPR/Cas9 based molecular recorder for storing developmental signaling cues as permanent mutations in cellular genomes for later readout by scRNA-seq. They apply SABER-seq to record Notch signaling in developing zebrafish brains. Molecular recording is an exciting emerging technology, and this manuscript constitutes an important step towards establishing powerful recording systems. However, the performance of the system presented here, while clearly going beyond the published state of the art, is still affected by considerable limitations regarding the dynamic range (binary readout), the temporal control (long induction needed), and the biological interpretation (see major points 1 and 2). While these limitations certainly restrict the biological application range, I would consider this manuscript to be suitable for publication in Development, since it is not only one of the first manuscripts to explore molecular recording of signaling cues, but it also includes careful characterization and calibration of the system. The following comments may help improve the manuscript further.

We sincerely thank the reviewer for their positive comments and suggestions for improvements. We agree that this work is an important step towards molecular recording of signaling cues during development. This study successfully establishes the genetics, experimental protocols and bioinformatics to do this.

We would like to offer some clarification based on the reviewer’s summary. SABER-seq is designed to tag cells that experienced a signal i.e. the goal is to associate Cas9 expression and subsequent activity with a triggering signal input. We demonstrate the first half by inhibiting Notch signaling and showing the number of Cas9-GFP fluorescent animals decrease (Fig. 2J) and the second half by confirming that barcode editing is impeded under these conditions (now Fig. 2K, previously Fig. S2d). These are significant advances as no other genetic system published to date has shown that Cas9 expression and activity can be regulated by in vivo signaling in a developing animal. As such, SABER-seq performs as it was designed, and these advances are valuable to the community. Measuring which cells received higher levels of signal than others is most certainly an exciting and important question but requires an additional layer of genetic control (with respect to double strand break repair) beyond what we initially designed SABER-seq to accomplish. SABER-seq is not

© 2024. Published by The Company of Biologists under the terms of the Creative Commons Attribution License (https://creativecommons.org/licenses/by/4.0/).

meant to replace all existing signal tracing methods but provide an alternative where transcriptomics and high throughput data would be beneficial. We propose the current version of SABER-seq can be used in conjunction with established methods such as fluorescence imaging to differentiate high vs low Notch activity in cells of interest. We have now emphasized these points in the Introduction and Discussion considering this reviewer's comments.

We use a long induction period to capture the full spectrum of cell types that have the capacity to respond to Notch within a tissue of interest. That is, by increasing the total duration of CreERT2 induction, we increase the overall recording period and tag more cells. This goal is also now clearly stated in the Introduction. We do obtain edited barcodes with a single 30-35 min heat shock treatment (Fig. 2I) but since the duration of recording is reduced, we detect editing in a smaller fraction of cells. Please see new data in Fig 4E and 4F, and comments to Point 2 as support. If a user wishes to sparsely label Notch active cells, they may reduce the number of heat shock cycles.

Reviewer 1

Comments for the Author:

Major comments:
1) An interesting application of such a recording system would be to relate signal exposure in specific time windows to the resulting cell fate decisions. This question is only addressed at the very end of the manuscript, in Fig. 5d. This part should be expanded. In particular, as presented, it is not clear how big the differences between different cell types are in terms of the fraction of edited barcodes.

We have re-written and expanded the relevant Results section (now Fig. 6) and modified the Discussion. Please refer to the new text and figures. It is important to note that although Notch signaling has been widely shown to directly regulate cell fate and specification of neuron subtypes in *Drosophila*, the same is not true for the vertebrate nervous system. Only a limited number of studies have shown that Notch signaling controls the identity of a select few neuronal subtypes in the brain, eye and spinal cord of vertebrates (reviewed in PMID 19799767 and PMID 21382546). For example, until recently there had been no evidence for a role of Notch signaling in binary neuronal cell fate choices during mammalian brain neurogenesis (the exception being cerebellar progenitor cells that regulate Purkinje cell vs granule cell fates via Notch; Zhang 2021 PMID 34107249). Instead, the predominant role of Notch in the vertebrate brain, particularly during early development, is to maintain progenitors and prevent their differentiation into any neuronal fate. As such, the expectation from SABER-seq data in our experiments would be that there are no significant differences in the fraction of edited barcodes between neuronal subclusters. This is what we observe. We note 3 neuronal subclusters where the edited barcode fractions were statistically different. One of them is a Purkinje cell subcluster with 100% edited barcodes across 3 brain samples. This is consistent with the finding in the mammalian cerebellum where high Notch activity was required for Purkinje cell generation from $Sox2^+$ cerebellar progenitors (Zhang 2021 PMID 34107249).

2) It looks as if the fraction of edited barcodes does not vary much between the different cell types of the brain. Since one main focus of this manuscript is to introduce a novel methodology, it would be important to also analyze another tissue (or another period of induction, or another stage of the fish) in which there is a clearer relationship between signal exposure and cell type. This would be required for a proof of principle.

We hope we are interpreting the concern of the reviewer correctly. To the best of our understanding, it appears that the reviewer is asking since the brain is a tissue where barcode editing is detected in nearly all cell types, what is the control to ensure editing is due to cell exposure to Notch signal (similar to the concern raised by Reviewer 3). Please see our response to Reviewer 3 Comment 2 for how we demonstrate Notch-dependent Cas9 activation and subsequent barcode editing. We chose to record Notch in the brain precisely because it is so widespread and critical for general neurogenesis. This allows us to calibrate the Notch sensor and recorder and benchmark the barcode editing rates. These are now stated in the Introduction and Results

© 2024. Published by The Company of Biologists under the terms of the Creative Commons Attribution License (https://creativecommons.org/licenses/by/4.0/).

sections.

To address the reviewer's suggestion of analyzing barcode data from another tissue, we analyzed barcode editing from several non-central nervous system tissues at 3 wpf and adult stages using PCR and sequencing (new Fig. 3). Barcode edited fractions are highest in brain and eye tissue samples, and lowest in fin, skin, jaw and skull tissues. These results confirm that the *her4.3* promoter is activated by Notch in fewer non-central nervous system cells during development compared to the brain. To address the reviewer's suggestion of analyzing barcode data from another period of induction, we performed a single round of heat shock treatment at 8 hpf and analyzed barcodes at 3 wpf (new Fig. 4E, F). This revealed a lower level of barcode editing in the brain (22% with 1 heat shock cycle vs 83% with 17 cycles of heat shock) and differences in the nature of barcode edits. This demonstrates that the fraction of edited barcodes changes with the duration of induction/recording.

3) In Fig. 2i, the authors demonstrate that heatshock cycling is necessary to achieve high levels of editing. Does this rather harsh treatment have any negative effects, e.g. developmental defects or increased mortality? Can you pleases describe how you tested for adverse effects or how you prevented them from occurring?
Also, can you please specify if the heatshock cycling was performed in the same way (and for the same duration) in the 3 dpf and 3 wpf samples? Observing strong editing after the end of induction would be a more direct argument for ongoing recording than continuous detection of GFP (as discussed in line 197).

We observed no significant developmental defects or increase in mortality during heat shock cycling (new Fig. S2E). The animals are quite resilient by the time cycling is started at ~24 hpf. There is also a 45 min recovery period after each heat shock. Heat shocked animals were raised to adults and showed no readily observable changes in their gross anatomy, overall growth, time to sexual maturity, fertility or life expectancy.

Heat shock cycling was performed in the same way and for an identical duration for all animals (3 dpf samples and 3 wpf). In fact, after performing heat shock cycling, we simply raised a subset of fish to 3 weeks or sacrificed a subset at 3 days. 3 dpf samples are generally collected as a calibration test to ensure our crosses and treatments were successful prior to data collection at 3 wpf. We observe strong editing shortly after the end of the heat shock period when we test for barcode editing at 72 hpf but we have not tested if the edited fraction continues to increase beyond 72 hpf.

Minor comments:

4) It would be good to state explicitly already at an earlier stage in the manuscript that the dynamic range is limited and that you go for a binary analysis. Otherwise the reader might worry about the tandem repeats in the SRE (non-linearity), Cas9 excisions of previously recorded events, or other features that could reduce resolution, but are not of major importance for the current binary readout.

Thank you for the suggestion. We have modified the Introduction to state this goal.

5) Fig. 5b: Maybe you can consider highlighting not only the cells with detected SABER barcode, but also highlight those with an edited barcode in a different color. Right now, the statement in line 317 ("Accordingly, we detect edited SABER barcodes in neural progenitors, immature neurons and mature neurons (Fig. 5b)") is not really apparent in the figure. Also, I found the statement in the figure caption that cells with barcodes are shown in red confusing, since not all of these dots appeared in red in the pdf (this might be a result of figure compression).

We have replaced the figure panel with one showing the unedited and edited barcodes in different colors (Fig. 6C).

6) Guide RNA targets: Could the authors please clarify the targets of the guide RNAs in the experiments described in figure 1? In lines 118-120 the authors mention inclusion of a guide RNA against tyr – is this guide RNA one of the ones shown in figure 1b? Additionally, is the guide RNA against tyr retained in the her4.3:switchCas9 fish, resulting in a total of five guide RNAs in those fish?

© 2024. Published by The Company of Biologists under the terms of the Creative Commons Attribution License (https://creativecommons.org/licenses/by/4.0/).

The sgRNA sequences in Fig. 1 and Fig. 2 are identical. The entire sgRNA cassette is the same for all transgenic lines described in the study (available as a destination vector for multi-site Gateway cloning). We simply swap out the promoter sequence (available as 5ƍ entry vectors for Gateway cloning). Thus, there are 4 sgRNAs for both *tp1* and *her4.3* reporters described Fig. 1 and 2, and for the *Olactb:Cas9-GFP* transgene in Fig S1. sgRNA 1, 2, 3 target the dsRed sequence. sgRNA 4 targets *tyrosinase (tyr)* sequence. The disruption of the stripe pattern in zebrafish adults shown in Fig. 1, S1 and S2 result from the activity of sgRNA 4 (targeting endogenous *tyr* locus). The remaining 3 dsRed sgRNAs are not used for analysis in Fig. 1 or S1 as there is no dsRed sequence to target. However, all 4 sgRNAs are used for analysis in Fig. 2 and S2 since the *SABER* barcode construct contains 3 dsRed target sequences and 1 *tyr* target sequence arranged sequentially in a concatemerized array. We have rewritten the text and modified the Figures to make this clear.

7) Lines 75-78: "In addition to lineage analyses, studies have applied molecular recorders to record a wide array of cellular and synthetic events such as gene expression, cellular stimuli, synthetic signals, videos, and text messages but these have been limited to cell culture systems or prokaryotic cells and have not been combined with scRNA-seq" - This sentence describes very different applications followed by eight sources. Maybe it's worth separating biological and technical applications to improve clarity?

Thank you for the suggestion. We have modified as requested.

8) Lines 302-304: Is there a specific reason the authors chose beta-actin to drive the recorder transgene?

Beta-actin is one of the strongest ubiquitous promoters available for zebrafish transgenics. We wanted to ensure high expression of the recorder in a wide variety of cells to facilitate detection by scRNA-seq.

9) Figure 3c: Are cells where the entire target is deleted included under the intersite category? Targets 2 and 3 seem to have been excised in some cases according to fig. 3d.

Yes, they would represent deletions of target sites 1-4 (green ribbon on the chord plot spanning Target 1 to Target 4).

Reviewer 2 Comments for the Author:

I very much enjoyed reading this manuscript. It demonstrates a creative and original solution to recording signalling activity during development. I found this work inspiring and I am confident other readers will too. The presentation and writing style are of high quality. This makes the paper more digestible, which is much appreciated as some of the genetics is not trivial. In my opinion, the paper could be published in Development with no edit. Here are some suggestions for the authors if they wish to further improve the paper.

We are pleased to know that the reviewer found our work inspiring and suitable for the *Development* audience. We sincerely thank the reviewer for their appreciation of our work and suggestions for improvements.

--- On the science ---

1– A potential pitfall of the approach is DNA repair not being equal in different cell types or across development or at different stages of cell cycle. Namely, I think the approach somewhat assumes that double-strand breaks (DSBs) are always repaired in the same way, but this is unlikely to be the case. While perhaps not a crucial consideration now, it would become important if making the approach more quantitative in the future, as suggested in Discussion. When Cas9 is injected at the one-cell stage, embryos mainly rely on alternative end-joining to repair DSBs (cf. Thyme 2016), but I doubt this remains the case for long as it is highly error-prone. Non-homologous end-joining, for example, is more accurate, i.e. leaves fewer indels. Accordingly, I think the sensitivity of SABER would (annoyingly) probably vary between different cell types or developmental stages? As in, a little pulse of Notch signalling in a cell that uses non-homologous end-joining may leave no trace

© 2024. Published by The Company of Biologists under the terms of the Creative Commons Attribution License (https://creativecommons.org/licenses/by/4.0/).

in the barcode, while the same pulse may leave indels in a cell that uses alternative end-joining. This point could be elaborated upon in Discussion, maybe around line 399.

This is a great suggestion. We have added a sentence describing this in the Discussion.

2- Silencing of Cas9 expression is a limiting factor, not only for this approach. Would insertion of the transgene in one of those pIGLET safe harbor loci help in any ways?

pIGLET safe harbor loci may help. We have not tried this method yet but will check this for future iterations.

3- About making the system more quantitative, maybe it is possible to find a hint of this possibility in the current data? My logic would be: one could probably estimate the number of double-strand breaks for each "barcode allele", giving a signal from 0 to 4. Now, if we can identify a few cell types which are known to have received subdued/brief Notch signalling, do they tend to have lower number of double-strand breaks? What about cell types with continuously high Notch signalling, do they all have alleles which are consistent with 4 double-strand breaks (e.g. small indel at each site)? Maybe some version of this idea is the way to go for quantitative recording? You would probably want to prevent large deletions somehow (as e.g. a large deletion between target 1 and 4 makes it impossible to count the 'real' number of double-strand breaks, anywhere from 2 to 4), maybe with that DNTT polymerase or inhibition of alternative end-joining (see inhibitor ART-558, but ideal would be fusing some inhibitor to the Cas9 from the transgene).

Thank you for the suggestion. The number of edits in most recovered barcodes is closer to 1 rather than 4. Thus, filtering the dataset to barcode with 4 indels would result in insufficient barcodes for statistical testing. We agree that minimizing or preventing large deletions would be a step towards making the system quantitative (e.g. using DNTT as mentioned in the Discussion).

4- To record multiple signals, would it not be possible (and much easier/scalable than expressing different Cas proteins) to simply express different gRNAs and have a bigger barcode with more targets? Maybe there is a simple reason why this does not work...

Expressing up to 5 sgRNAs from a single plasmid construct using U6 promoters has been demonstrated in zebrafish (Yin 2015 PMID 26285757), however expressing more than 5 has not. It may be possible to split the design across two constructs to produce 10 sgRNAs. However, since all the sgRNAs are under constitutive U6 control it is not possible to make them responsive to different signal inputs. Switching to a tRNA or ribozyme multiplexing system to separate many sgRNAs and using a signal-specific Pol II promoter to drive the entire construct may work. In our hands, we weren't successful in obtaining sgRNA expression using either of these approaches.

5- There are many steps before the first indels occur, in chronological order: Cre transcription + translation, Lox-P excision, Cas9 transcription + translation, assembly with sgRNA, double-strand break, repair (probably multiple cycles of break & repair as the repairs may be correct most of the times). Any guess on how long it all takes? This would impact experimental designs/interpretation of results, e.g. it might be that there is a 6-hour lag between start of recording and first indels. I guess you could fairly easily test this by starting recording then extracting genomic DNA at successive timepoints. How early can you detect indels?

We do not have a definitive answer to this question. With a single heat shock cycle at 8 hpf, we can detect edits at 24 hpf. We have attempted to determine if edits appear earlier but the genetics of the cross make this quite difficult to perform cleanly as we are unable to screen embryos that are double positive for SABER and Cas9 until ~24-36 hpf.

6- Results in Fig. S2: I am not sure I understood the pigmentation defects in those fish. In my understanding, the full SABER system does not have a gRNA targeting tyr anymore, as that was

© 2024. Published by The Company of Biologists under the terms of the Creative Commons Attribution License (https://creativecommons.org/licenses/by/4.0/).

just to test the system and we do not want to create mutations in endogenous sequences?

The fish do have *tyr* sgRNA. The sgRNAs used in all experiments are the same. Please see comment to Reviewer 1, Point 6. The SABER barcode contains a corresponding *tyr* target sequence downstream of the 3 dsRed target sequences.

7- I was slightly confused by the transition to the section starting at line 306. Do I understand correctly that 126/127 of cell clusters had edited barcodes? It sounds a bit odd to check whether they are the "expected" cell types if that is (virtually) all of them and there is no negative control, so to speak. What is this one cluster that did not have edited barcodes? Maybe that is interesting?

We agree the statement was confusing and have removed it. The one cluster with no edited barcodes is a radial glia cluster from which we only retrieved 1 cell with a barcode. Due to this low sampling, we only recovered an unedited barcode and no edited barcode.

--- On the writing etc. ---

8- Fig. 2i: I would not label the two bands "edited" and "unedited". The majority of edits may well be small indels (this is certainly the case when injecting one gRNA), which would not be easily detected on the gel.

We have removed the "unedited" label.

9- line 62, I see this is common usage, but I am slightly confused by "single-cell genomics". I think we are mainly referring to transcriptomics here? I would understand single-cell genomics as sequencing genomes of single cells. I think "single- cell sequencing or transcriptomics" would be better.

We have revised to single-cell sequencing.

10- I think the writing around 168–173 could be a bit more explicit to help readers. Something like: "to trigger … excision of the dsRed and stop codon, allowing Cas9 expression"; then "GFP expression in the brain, indicating successful excision of the dsRed and Cas9 expression".

Thank you for the suggestion. We have modified as stated.

11- Around 184–188, I would use the transgene names introduced before? e.g. "single transgenic" is SABER, right?

Yes, that is correct. We added the transgene names as suggested.

12- Line 205: "switching on Cas9-GFP expression when Notch activity is inhibited", I was slightly confused by this wording. In theory, Cas9-GFP expression should be off when Notch is inhibited. How about "switching on recording when…"?

Thank you, we have modified the wording.

13- Results & Figure legend: stripe "mutations", I would say "defects" or else? To keep mutations as meaning DNA mutations.

We have modified as stated.

© 2024. Published by The Company of Biologists under the terms of the Creative Commons Attribution License (https://creativecommons.org/licenses/by/4.0/).

Reviewer 3 Advance Summary and Potential Significance to Field:

This work presents a new method to record developmental signals. A notch-responsive CRISPR/Cas9 makes edits to a barcode (in this case 4 sgRNA sites), and the edits are sequenced alongside single-cell RNA-sequencing to determine whether the cell experienced notch signaling. In addition to this tool, a new atlas of the 21-23 dpf zebrafish brain (almost 150k cells) was included. This is a well-written manuscript with clear figures and presentation. Conceptually, the tool is interesting, and I commend the authors on making progress on this interesting technical challenge. There are, however, some aspects of the tool and conclusions that need further validation. Given the technical issues and questions that remain, it is unclear whether it would be adopted by the field when other options exist. The biological demonstration of the tool, more or less editing in cell types that receive differential notch, is also not sufficiently validated. Finally, while the new scRNA-seq data is itself extremely valuable to the community, there are issues with its annotations and the depth of analysis completed. Building a new atlas is obviously not the goal of this work, but publishing inaccurate annotations could lead others in the field astray.

We sincerely thank the reviewer for their constructive suggestions and recognizing our progress in developing a new tool.

Reviewer 3 Comments for the Author:

Major comments

1) While I understand there is novelty in using CRISPR, as well as the possibility to scale the concept with orthogonal systems, the method still feels somewhat preliminary. One main issue is that only seems able to measure the on/off state of signaling, or at least there was no attempt to determine whether edits to multiple sites in the barcodes had any meaning as far as signaling level. The concept presented is that the number of cells in a cluster that are edited is a proxy of signaling for that cluster. That is possible, but it would be much better to be able to have some idea of signaling level. This is a first pass at a challenging problem, so the method is still worth considering despite this point. There are also other challenges that came up, though, with only up to 20% of the barcode sequences even being recovered using this method. Given the experimental challenge of needing to recover the DNA barcode separately from the RNA sequencing, and that it is not working very well, I wonder if this approach is really a good way to accomplish the research goal. Since an on/off signal is all that is desired, perhaps measuring the RNA from a very stable and highly-expressing promoter would be better. For example, making Cre notch-responsive instead of Cas9, and then flipping on expression of a stabilized RNA that is under control of b-actin, could be an alternative that might be less technically challenging and give better recovery of the signal. With the current success of the method, I am left wondering why this is a good idea compared to alternatives.

As the reviewer has noted, this work represents a foundational step towards building a more comprehensive toolset for tagging cells exposed to a signal of interest. Our main performance goals for this iteration were to ensure resolution, throughput and long-term genomic storage of a past signaling event. All of these constitute advances over other currently available methods. Single-cell resolution is achieved with scRNA-seq. Throughput is achieved due to use of a droplet-based scRNA-seq platform. Permanence is achieved due to genomic barcoding. Although there certainly is room for improvement in barcode recovery, the high throughput nature of the technique enables recovery of large numbers of signaling events simultaneously with cell identities. This underscores the power of SABER-seq (scalability). Recovery issues have come up in other barcoding technologies as well. For example, scGESTALT had a recovery of 6-28% initially (Raj 2018 PMID 29608178) prior to optimization to 65% in the second generation (Raj 2020 PMID 33068532). CARLIN had a recovery of 10% (Bowling 2020 PMID 32413320) prior to improvement to ~70% in DARLIN (Li 2023 PMID 37852258). Furthermore, droplet-based scRNA-seq itself suffers from significant sample loss due to tissue dissociation resulting in the absence or limited representation of rare cell types in the datasets. However, all these methods rely on profiling several thousands to millions of cells to ensure coverage. If the profiled tissue is very precious with limited availability (i.e. low sampling power), researchers should use alternative signal profiling tools. Taken together,

© 2024. Published by The Company of Biologists under the terms of the Creative Commons Attribution License (https://creativecommons.org/licenses/by/4.0/).

SABER-seq is a powerful approach when transcriptional profiles and throughput are desired.

We appreciate and understand the desire to measure signal levels but there exist many applications where this level of quantification is not required. For example, our method would be useful for generating richly annotated “cell trees” where lineage, signaling, epigenetic and transcriptional measurements of cellular states are embedded along various branches of the tree (discussed in McKenna and Gagnon 2019 PMID 31249005, Domcke and Shendure 2023 PMID 36931241). Our method is also useful for performing genetic perturbations (e.g. crispants of signaling components) and assessing overall effects on cell population. A similar form of “high dimensional phenotyping” has been presented in Saunders 2023, PMID 37968389. Moreover, by making SABER-seq compatible with 10x scRNA-seq, users can take advantage of existing 10x assays to obtain multi-modal measurements from a single experiment. Additional biological insight can then be gained on specific lineages with traditional approaches measuring signaling strength using imaging-based techniques. To the best of our knowledge, there isn’t a high throughput, high resolution approach compatible with multiplexing assays that would serve as an alternative. We have adjusted our Discussion to emphasize these applications and benefits.

The alternative genetic approach the reviewer has suggested is also interesting. However, its feasibility hasn’t been demonstrated so we find it difficult to offer a point-by-point comparison of its efficacy compared to SABER-seq. One issue we foresee is that inducible control of Notch recording will be compromised as leaky CreERT2 expression would result in some conversion as soon as the Notch-active promoter is expressed. In our system, since CreERT2 is separated from Notch activity, we have better control over when exactly Notch recording is initiated. As shown in Fig. 2H and 2I (lane 1), we do not detect conversion or barcode editing outside of our treatments. Furthermore, in our experience, any transgenic mRNA (even “stable mRNA” e.g. GFP from b-actin promoter) would still require a separate cDNA library amplification step to enrich the amplicon since there is strong variability in mRNA representation (i.e. dropouts) in scRNA-seq data. Finally, our approach allows for “future proofing” the recording system for multiplexed measurements using alternative Cas proteins.

The assumption that higher signals would result in more discrete (small) barcode edits is too simplistic based on our experience with Cas9 barcode editing. More signaling could (and likely does) cause several cutting events but how the cell repairs those cuts is more complex (please see Reviewer 2’s assessment of the repair complexity in comments 1 and 5). The cell often generates a large deletion that spans all the individual cuts (inter-site) rather than repairing them to give small individual indels (intra-site). Furthermore, previously stored small indels can be erased with 1 larger deletion if Cas9- induced cutting continues for extended periods. This has been observed by us and others in barcoding applications where targets are arranged in a tandem array (Raj 2018 PMID 29608178, Bowling 2020 PMID 32413320, Li 2023 PMID 37852258). It is similar to the concept of generating null alleles using multiple sgRNAs spanning a large targeting sequence. Interestingly, when recording Notch with a single pulse of Cas9 activation we observed fewer inter-site deletions (new Fig. 4F), likely because Cas9 is active for a shorter period. Thus, although more signal may result in more Cas9 expression and activity, the cellular repair would favor large deletions. Therefore, the number of edits in most recovered barcodes is closer to 1 rather than 4. This makes deducing signaling levels by counting how many discrete edits are found per barcode not a valid form of analysis. As we mentioned in the Discussion, switching to a different form of barcoding e.g. with Cas9-DTT fusion may mitigate this issue. Another idea would be to use a highly destabilized Cas9 (Cas9 fused to a degron) to favor one cut per double strand break repair period. Other options include using multiple independently integrated barcodes with 1 targeting sequence per barcode (i.e. dispersed barcode arrays rather than one concatemer array) or using prime editing and pegRNAs to count signaling activity on a DNA “ticker tape”. Each of the above has its limitations that will require additional testing and design modifications to solve.

2) My biggest concern is regarding whether the edits observed in the sequencing are really from notch-responsive events. The her4.3 promoter is supposed to be responding to notch, particularly in the development of the brain. The edits appear to be equally distributed, which is mentioned by the authors, and even appear to be found in blood cells. There are also pigment disruptions in the her4.3 promoter fish, which makes sense given the presence of edits in every cluster. The supposition that these edits are due to notch signaling rests heavily on the single gel in Figure 2i. Additional information added to that gel would be helpful: what do the editing profiles look like in

© 2024. Published by The Company of Biologists under the terms of the Creative Commons Attribution License (https://creativecommons.org/licenses/by/4.0/).

non-neuronal tissues at 3 wpf and what does the control look like at 3 wpf? The gel is clean and fairly convincing, but the presence of these edits throughout so many non- neuronal cell types is not consistent with what we know about her4.3 expression. It is possible that it would be transiently expressed in those types, but then I think that one would expect a lower level of editing compared to types with much more sustained expression. The discussion states that "her4.3:switchCas9 was expressed in ancestors of melanophores". There are ways to check this point, as well as the other non-neuronal cell types that display unexpected editing, such as imaging of their existing switch reporter line in combination with HCR to label cell types. Even if the GFP signal degrades, there should still be a sufficient time window to determine whether the lox sites are being edited outside of the brain as well.

We are unsure if the reviewer is questioning whether the edits observed by sequencing are due to sequencing errors or if the Notch-responsive Cas9 transgenic line we are using is not truly Notch-responsive. We explain both situations below.

A possibility may be that sequencing errors or library preparation errors result in the appearance of edited barcodes in our data. However, in a previous publication where we used Cas9 barcoding for lineage tracing, we show via sequencing that the frequency of editing is <0.1% in libraries from samples where Cas9 and sgRNAs are not expressed (Raj 2018 PMID 29608178). Furthermore, in this study we use two barcode-specific primers to selectively amplify the barcode from the transcriptome (via nested PCR) when preparing libraries. During analysis we also filter out any reads that do not start with the barcode-specific primer sequence.

We assume that barcode editing occurs due to Notch signaling because Cas9 is under *her4.3* promoter control. For barcode editing to occur, Cas9 needs to be expressed. Cas9 is only expressed when the *her4.3* promoter is active and after CreERT2-mediated excision of the upstream loxP cassette. There is no other source of Cas9 in our genetic system. *her4.3* has been established and used as a Notch activity reporter in many published studies since 2007. The promoter has been validated using luciferase assays and fluorescence imaging in gain-of-function and loss-of-function experiments. It has 3 confirmed Rbpj binding sites (the signaling response element), which represent co-factors of the Notch signaling response. In our first submission, we provided three lines of evidence to further confirm Notch dependence. We show (1) Notch inhibition results in decreased *her4.3* activity as measured by lower fluorescence, Fig. 2C, 2D. (2) Notch inhibition results in fewer Cas9-GFP positive animals, Fig. 2J. (3) Sustained Notch inhibition results in no barcode editing, now Fig. 2K (previously Fig. S2D). Please also see this reviewer's comment in Point 11 below where they agree that "…the editing is notch responsive". Moreover, we show Cas9 expression is tightly regulated by CreERT2 expression (Fig. 2H), and in the absence of 4-OHT there is no barcode editing, Fig. 2I lane 1. To further strengthen this aspect, we have added additional data. We show that crossing her4.3:GFP (no Cas9, no sgRNA) with the SABER barcode line does not result in barcode editing (new Fig S2G). Similarly, crossing her4.3:loxP-dsRed-loxP-Cas9-GFP (no sgRNA cassette) with the SABER barcode line does not result in barcode editing, new Fig S2H. These results rule out spurious/spontaneous barcode editing in vivo. Furthermore, as described in Reviewer 1 Comment 2, we show that a single heat shock pulse at 8 hpf results in 22% barcode editing in 3 wpf brain compared to 83% editing when we perform 17 heat shock pulses (new Fig. 4E). In summary, we show (a) without Cas9 there is no editing, (b) without sgRNAs there is no editing, (c) without 4-OHT + heat shock there is no editing, (d) with Notch inhibition there is no editing, and (e) editing levels increase with increased duration of Notch recording. Collectively, these results and past studies validate use of the *her4.3* promoter to drive Cas9 in a Notch-dependent manner.

There may be a misunderstanding regarding pigment disruption due to the way we phrased the sentence in the Discussion. We apologize for the oversight. Pigment defects are not observed in the *her4.3* promoter fish (i.e. *her4.3:switchCas9*). These fish have normal stripes. Defects are detected in the adult F2 progeny resulting from crossing *her4.3:switchCas9* x *SABER* F1s and performing heat shock and 4-OHT treatment. That is, they appear after Notch- dependent Cas9 activation. Additionally, pigment disruptions in these adult offspring are minor compared to the *Olactb:SpCas9-GFP, 4[U6:sg]* transgenic where Cas9 is not under Notch regulation and is ubiquitously expressed (compare to Fig S1B). They are also less prominent relative to the *tp1:Cas9-GFP, 4[U6:sg]* transgenic (Fig. 1C) where Cas9 is under Notch regulation but is not under inducible control. The *tp1* element has been validated in zebrafish, mouse and cell culture as Notch dependent and is

© 2024. Published by The Company of Biologists under the terms of the Creative Commons Attribution License (https://creativecommons.org/licenses/by/4.0/).

more widely active than *her4.3*. Additionally, *tp1* is active in neural crest cells, the ancestors of pigment cells. Thus, based on our data we propose that the *her4.3* promoter may be active, at least transiently, in ancestors of pigment cells or pigment cells themselves. Indeed, scRNA-seq data from a recent zebrafish atlas study (Sur 2023, PMID 37995681) shows expression of *her4.3* in the xanthophore pigment lineage in early development (new Fig. S2C). Furthermore, although *her4.3* is widely studied in the context of neurogenesis, it is not exclusively expressed only in neural cells. Indeed, past studies have described expression and activity in the hypoblast, presomitic mesoderm, neural crest cells, and fin blastema (please refer to text modifications that highlight these). Thus, observing barcode editing in some classes of non-neural cells is not unexpected and is in fact an advantage of the resolution power afforded by combining signal recording with scRNA-seq.

As requested, we have included new data for barcode editing from non-neural tissues (fin, jaw, skull, skin) from 3 wpf and adult stages (new Fig. 3). Barcode editing was enriched in the brain and eye tissues compared to the non-neural tissues profiled. We observe as much as a 100-fold decrease in edited fractions between the brain and non-CNS tissues. In light of all this evidence, we are confident that our data unambiguously represents barcode editing resulting from *her4.3* promoter activation due to Notch signaling.

The reviewer states *"…determine whether the lox sites are being edited outside of the brain as well."* We do not fully understand this comment. The loxP sites upstream of the Cas9-GFP cassette in *her4.3switchCas9* transgene are most likely excised in all/many cells (not just brain) of fish resulting from *her4.3switchCas9* x *SABER* cross since CreERT2 expression is ubiquitous when induced (driven by heat shock promoter). We do not suspect spontaneous loss of loxP in the absence of CreERT2 function. As stated above, the only source of Cas9 expression would be if the loxP sites are deleted AND *her4.3* promoter is active. Both these conditions are only satisfied when *her4.3switchCas9* is crossed with *SABER*, resulting embryos are treated with heat shock and 4-OHT, and *her4.3* is activated by Notch.

3) The point about identifying possibly different notch responsiveness in the TL/GC subtype compared to the Purkinje type is interesting. It is, however, not very convincing in light of the previous point about the edits being everywhere regardless of how likely it is for her4.3/notch to be involved in their development. In the condition of notch inhibition over the identical time window used for these editing experiments, are certain cell types more or less likely to be impacted? An HCR following inhibition could give insight into this point. Also, on a related point, approximately half of the barcodes were recovered from each of two experiments. Were there any differences in which cell types had barcodes in each experiment (such as this 5D finding, did it replicate in both experiments)? It is probably possible to come up with a biological system where it is known that notch is not required in some cells and not in others, and that may be an option for a biological demonstration of the tool's effectiveness.

As noted in the response above, we have added several lines of evidence to demonstrate that the barcode editing we observe is Notch-dependent and enriched in the brain. Please also refer to our response to Reviewer 1 comment 1. Although Notch signaling has been widely shown to directly regulate cell fate and specification of neuron subtypes in *Drosophila*, the same is not true for the vertebrate nervous system. Instead, the predominant role of Notch in the vertebrate brain is to maintain progenitors and prevent their differentiation into any neuronal fate. Our finding that a Purkinje cell subcluster had 100% edited barcodes is consistent with the finding in the mammalian cerebellum where Notch activity was required for Purkinje cell generation from $Sox2^+$ cerebellar progenitors (Zhang 2021 PMID 34107249). Yes, we recovered barcodes from all 3 brains for the clusters that showed significant differences in the fraction of edited barcodes (TL_2, GC_3, PurN_6), thus our results replicated across all experiments. This is now indicated in revised Fig. 6D, 6E. The purpose of this tool is to show that we can successfully record Notch activity. In the brain, the expectation is that all neuron subclusters would inherit edited barcodes as the ancestral progenitors were subject to developmental Notch signaling. Our data clearly shows this is the case. Contrast this with non-neural tissues where barcode editing is rarer (Fig. 3). Therefore, we believe we have demonstrated the tool's effectiveness.

Per the reviewer's suggestion, we performed additional experiments where we pharmacologically inhibited Notch (using LY-411575) from 4-72 hpf, which overlaps the time

© 2024. Published by The Company of Biologists under the terms of the Creative Commons Attribution License (https://creativecommons.org/licenses/by/4.0/).

window we used for recording Notch activity in the SABER-seq experiments (new Fig. 6F-6H). We note that this treatment is quite harsh and results in lethality shortly after 72 hpf. We confirmed suppression of Notch by decreased *her4.3* mRNA expression via qPCR. We observed a general trend of decreased expression of various neuronal markers during this treatment, suggesting that early and prolonged Notch inhibition impairs overall neurogenesis by 3 dpf. This is not surprising since Notch inhibition decreases late differentiating (secondary) neuron populations born after 2 dpf due to excessive loss of neuroepithelial progenitor cells (Jiang 1996 PMID 9007241, Bingham 2008 PMID 14579383), which subsequently give rise to radial glia cells. We also performed HCR using markers for Purkinje cells and granule cell neurons in control and drug treated animals. The overall sizes of both subtypes appeared to be reduced, however we observed gross brain morphology impairments, which made quantification of differences between these populations challenging. Since both these cell types are born after 48 hpf, it is difficult to resolve if loss of Notch signaling affects one more than the other in our inhibition assay due to widespread effects on secondary neuron populations when Notch is suppressed during early development. We propose a better experiment would be to selectively impair Notch activity in cerebellar progenitors and follow lineage progression (similar to experiments done in the mammalian brain, Zhang 2021) however this is beyond the scope of this manuscript.

4) I have significant concerns with some of the cell type assignments. For example, otpa/otpb are mainly markers of the peoptic area, not the subpallium (line 267). It is possible that further subclustering could resolve some of the issues. Other genes in this cluster (e.g., bsx) also indicate that it is preoptic area or that it requires further subclustering. Another example that is a little confusing is the galn in the TL. While I do see some potential literature on this, it is not reported in the zebrafish brain and larval in situ is not compatible with this idea. Third, the cluster labeled Hypothalamus 5 with high expression of synpr and sp8a lines up with a known subpallium cluster. What is the evidence that this cluster is from the hypothalamus? Fourth, Pallium_9 is strongly labeled by grm2a, which is an olfactory bulb marker gene. In summary, I would suggest making a detailed supplementary table with references to support each of the cluster annotations to support the point on line 259 that markers from the literature were used to assign types. Additionally, to support the point that previous datasets (references 36-38) were used for subregions, I suggest actually showing an explicit comparison of the clusters such as with a random forest plot. Additionally, there are also large brain regions not listed in the analysis, such as the tuberculum, thalamus, and hindbrain other than the granule cells (there are existing scRNA-seq datasets for this to compare to for this area). I suggest carefully going through the brain and cell type annotations to ensure their accuracy. I did not go through every cluster and proposed cell type, but I expect that there are more that either need justification or correction. Related to the comment above. Both dissected and whole brain data are included in the new scRNA-seq dataset presented here. However, no analysis was done to show the overlay of the dissected areas on those cell types, as far as I can tell. It would be very helpful to be able to match up the brain regions with the cell types, to further support their classifications. I understand that this is not the main point of the manuscript, but accurate annotations are important for any published dataset.

There are many points to address here. We have numbered them to make them easy to follow. The original Fig S3 is now Fig S4 and the original main Fig. 4 is now Fig. 5.

a) We have performed extensive brain cell type annotations in two published studies prior to this paper (Raj 2018 PMID 29608178, Raj 2020 PMID 33068532). We have also validated our method of cell type classification using in situs and smFISH (Raj 2020 PMID 33068532). Our classification method here is identical to our published methods. In our experience, we have found that many “marker genes” are found in several brain regions or cell types (at varying levels of expression) and using a combination of several markers is needed to reliably classify the clusters. This is why we have shown “shared markers” and “cluster specific markers” for all our dotplots. We have already performed 3-4 rounds of clustering and tried different resolutions for our analyses. We strongly suspect that performing additional rounds of subclustering would generate artifacts rather than capture biological differences. As requested, we have double checked our annotations and have updated as necessary (see d and e below).

b) *otpa* is indeed a marker of the preoptic area where it is highly expressed and found in

© 2024. Published by The Company of Biologists under the terms of the Creative Commons Attribution License (https://creativecommons.org/licenses/by/4.0/).

many cells. However, in Figure S4E the subpallium cluster we are referring to has a very low level of *otpa* (and even lower level of *bsx* - ~7% cells express it). We classify this as subpallium because the cluster also expresses *dlx1a*, *dlx2a*, *dlx5a*, *dlx6a* which are known subpallial markers. Additional domains of *otpa* expression beyond POA in the zebrafish larval brain have been documented including the subpallium (Vi region), posterior tuberculum, hindbrain and hypothalamus (summarized in von Bernhardi 2022 PMID 35708548), as well as a pallium cluster (Pandey 2023 PMID 37072188). It is possible that a few POA cells are clustering with this subpallium cluster (since the two regions are close together) but we were not able to resolve the cluster any further in a meaningful way. However, we realized that our original citation of Pandey 2023 PMID 37072188 for this cluster was incorrect. We have corrected it to the von Bernhardi reference in a new Table S3 and have removed *otpa* from the text and figure as it's not a strong marker of this cluster.

c) Similarly, we classified TL clusters because they express *neurod1*, *fat2*, *wu:fj39g12*. These markers have been shown to be expressed in the TL via in situ hybridizations in zebrafish adult brains (Takeuchi 2016 PMID 27615194). The authors state that the TL has shared molecular characteristics with granule cells in the cerebellum, which our clustering data supports (these structures always cluster closely). The other source of galanin cells that we are aware of is the hypothalamus/POA (Corradi 2022 PMID 35219430; Hiraki-Kajiyama 2024 PMID 38831653). However, the other markers in the cluster are not compatible with these regions.

d) Upon secondary inspection, we agree that the *synpr* and *sp8a* expressing cluster is likely part of the sub-pallium (Pandey 2023 PMID 37072188). We have updated the annotation (now SubPal_7). We also updated another prior hypothalamus cluster to sub-pallium (now SubPal_8). Since these regions are close in proximity with shared gene expression signatures, clusters from these regions are often closely associated. Additionally, we updated a former granule cell cluster (now Neuron_neto1l+_cntn4+).

e) After secondary inspection, we have updated the annotation of the grm2a+ subcluster to "Neuron_grm2a+".
Although *grm2a* is an olfactory bulb marker, we also observe expression of *tbr1b* (pallium marker) and *ebf3a* and *msi2b*, which are newborn neuron markers (Lange 2020 PMID 31908317). Thus, we are unsure if this is truly an OB cluster or represents a telencephalon cluster comprising newborn neurons. We have labeled it as Neuron_grm2a+ since we cannot confidently computationally assign a brain region to the cluster beyond generally belonging to the forebrain.

f) Structures such as tuberculum and thalamus are challenging to definitively classify in whole brain scRNA-seq data as we are not able to find a collection of stringent markers for these regions (Raj 2018 PMID 29608178, Raj 2020 PMID 33068532). We have instead labeled 5 midbrain clusters (other than TL) expressing *tcf7l2*, *barhl2*, *pax9*, *lef1* etc as MB 1-5, opting to leave them as generic midbrain annotations rather than wrongly classifying them. The diencephalon labels Di 1-7 likely contain thalamus, prethalamus, pretectum, etc. Hindbrain clusters are labeled in our dataset (HB 1-6, granule cells as the reviewer noted, Purkinje cells, upper rhombic lip [URL] progenitors).

g) As the reviewer noted we have included forebrain, midbrain, hindbrain scRNA-seq as well as whole brain scRNA- seq in this manuscript. However, this is not a new method of analysis. We have published the same strategy for 3 wpf brains in Raj 2018 PMID 29608178 where we did the exact analysis the reviewer requested. We compared the overlay of clusters from each of these brain regions with the whole brain datasets and matched them with markers described on ZFIN and other published literature to thoroughly classify the regions (can be found in Table S2 of Raj 2018 PMID 29608178). Nevertheless, we have added data in Fig S3 to show some examples of the overlay with the current dataset.

h) Since this is also a resource for community use, we have generated a Github repository which describes how users can download our processed Seurat R objects from GEO and run a ShinyApp script to visualize genes of interest in our dataset. We have also included a master script that includes all our clustering analyses with helpful descriptions of why we

© 2024. Published by The Company of Biologists under the terms of the Creative Commons Attribution License (https://creativecommons.org/licenses/by/4.0/).

performed certain steps. This will make it feasible for a user to run our data and perform their own analyses, including refining cell type annotations if necessary, in the future. We've mentioned this repository in the text. Here is the link for the reviewer's convenience: **https://github.com/BushraRaj- Lab/ZBrainAtlas_Siniscalco**

i) We have created a new Table S3 that includes references for our annotations.

Minor comments

5) Line 37: I am not sure I agree with the word "accumulates" in the abstract, given the emphasis later on about how this is an on/off label (edited/not edited). I think accumulates implies that the mutation count would increase with signal, which is not necessarily shown in this work.

We have removed the word "accumulates".

6) Lines 56-49: Why are imaging-based readouts limited to a predefined area? The entire brain can easily be imaged in zebrafish.

The whole larval zebrafish brain at 5-7 dpf (or earlier) can be imaged. However, this is not possible for juveniles or adults. We were also referring to the application in other systems e.g. mouse.

7) Line 163 (and possibly more): Check for the use of apostrophe versus the primer symbol.

Thank you, we have corrected this.

8) Line 199: Why is this data not shown? It seems like a useful point. I generally don't agree with including statements about findings if there is no data to support them.

Thank you. We were making the point that we observe GFP expression from 2 dpf to ~5 dpf. When we look at the larvae at 5 dpf, we see significantly less GFP signal. We have now added representative 2 and 5 dpf images showing a decrease in GFP signal (Fig S2F).

9) Line 221 and throughout: 10X is not correct (not large X).

We have corrected this.

10) Line 242/243: Seems not necessary in the Results section. The following sentence works fine as a topic sentence for that paragraph.

We have removed the sentence.

11) Figure 2: Why is Figure S2D in the supplement instead of along with 2i? It seems like a very important point (main text worthy) that the drug molecule reduces editing, showing that the editing is notch responsive.

We have moved the panel to the main figure as suggested (Fig. 2K). It appears the reviewer agrees that editing is indeed Notch responsive (in relation to their concern stated in comment 2).

12) Line 499: The plasmids are not currently on addgene, and the sequences are not provided as part of the manuscript supplementary information. While it mostly does not matter, the spacing between the guide RNAs and their sequences are not provided. This information would have been helpful for the review, such as for interpretation of the sequences in Table S1.

We have sent our plasmids to Addgene, they should be available after undergoing QC. The sgRNA target sequences on the barcode recorder are arranged in tandem with a 3 bp spacer between each target site. The barcode design is similar to our previous published work Raj 2018

© 2024. Published by The Company of Biologists under the terms of the Creative Commons Attribution License (https://creativecommons.org/licenses/by/4.0/).

PMID 29608178, and also similar to the barcode design in McKenna 2016 PMID 27229144. We have included the SABER barcode array sequence in Table S5.

13) Figure 4A: The colors do not appear to line up with the labels. Epithelial cells is supposed to be brown and pericytes are missing.

Our apologies for the oversight. These have been corrected.

14) The tab in Table S2 labeled PurN is empty, and the PurN neurons are in the Progenitors tab.

Thank you for pointing this out. These have been corrected

15) Table S2: What does a cluster label of “removed” mean. Why was this cluster removed?

This refers to a “cluster” that was removed from further analysis once marker gene analysis revealed no enriched markers. Thus, it appears this is not a true biological cluster but perhaps represents some technical differences.

16) Figure S3A: hypothalamus is misspelled. Why are the hypothalamus and diencephalon separately labeled? The diencephalon contains the hypothalamus. Also, why are some labeled and not others. There doesn’t appear to be a lot of reason for what is labeled and not, and it’s hard to tell what the cluster the label is referring to.

Yes, the diencephalon contains the hypothalamus but it also contains other structures (see comment to Point 4). The hypothalamus is easy to classify as a discrete sub-structure as it contains many highly expressing markers that have been validated in several studies. For clusters that we can confidently assign to a particular structure, we label them with the name of the structure. For clusters where we only know the broad section of the brain they came from (e.g. telencephalon, diencephalon, hindbrain), we label with that brain region. This is the annotation strategy we have already published in our brain scRNA-seq studies (Raj 2018, Raj 2020).

We have replaced the panel in Fig S4A (previously Fig S3A) with one that only includes the cluster numbers since the full labels are shown in Fig S4G.

17) Check the entire manuscript for appropriate italics on gene names.

Thank you, these have been corrected.

18) I was not given a reviewer token for the data on GEO. While this may not be the fault of the authors, it will be critical to ensure that all data is available on GEO and clearly marked before publication.

We are happy to provide a reviewer token for GSE268356. It is: ofipoeiazhazruh All the raw sequencing data, processed R objects, gene expression matrices and other processed files are available on GEO. We have an immaculate track record of being thorough with uploading and sharing our sequencing and analyses files on GEO and Github for all our studies (Raj 2018 PMID 29608178, Raj 2020 PMID 33068532).

Second decision letter

MS ID#: DEVELOP/2024/203102

MS TITLE: Barcoding Notch signaling in the developing brain

AUTHORS: Abigail M Siniscalco, Roshan Priyarangana Perera, Jessie E Greenslade, Hemagowri Veeravenkatasubramanian, Aiden Masters, Hannah M Doll, and Bushra Raj

© 2024. Published by The Company of Biologists under the terms of the Creative Commons Attribution License (https://creativecommons.org/licenses/by/4.0/).

ARTICLE TYPE: Techniques and Resources Article

I am happy to tell you that your manuscript has been accepted for publication in Development, pending our standard publication integrity checks. Please see comments from reviewer 3 which suggest optional addition of 2 sentences about possible caveats of cluster annotation and explanation for editing in unexpected cells. I don't view these as critical but they may help the readers to better understand your study. If you decide to add in 1 or both sentences please let the Development office and myself know with tracked changes. Alternatively or additionally, I encourage you to publish the peer review with your paper which will allow readers to see this suggestion.

Reviewer 1

Advance summary and potential significance to field

The authors have addressed all my concerns in a fully satisfactory manner, and I do not have any further comments.

Comments for the author

I recommend the manuscript for publication without further changes.

Reviewer 3

Advance summary and potential significance to field

This work presents a new method to record developmental signals. A notch-responsive CRISPR/Cas9 makes edits to a barcode (in this case 4 sgRNA sites), and the edits are sequenced alongside single-cell RNA-sequencing to determine whether the cell experienced notch signaling. In addition to this tool, a new atlas of the 21-23 dpf zebrafish brain (almost 150k cells) was included. This is a well-written manuscript with clear figures and presentation. Conceptually, the tool is interesting, and I commend the authors on making progress on this interesting technical challenge. The new scRNA-seq data is itself is also extremely valuable to the community.

Comments for the author

I appreciate the extensive experimental additions to the manuscript, in addition to the modified discussion. The manuscript is much improved. I have a couple of final comments. They are not critical, as all concerns were carefully addressed.

1) While I am not going to extensively check each of the clusters with the literature, I would like to point out that point “e” in the rebuttal indicates that there may still be some work to be done to increase certainty about the annotations. The authors state the following: “After secondary inspection, we have updated the annotation of the grm2a+ subcluster to “Neuron_grm2a+”. Although grm2a is an olfactory bulb marker, we also observe expression of tbr1b (pallium marker) and ebf3a and msi2b, which are newborn neuron markers (Lange 2020 PMID 31908317). Thus, we are unsure if this is truly an OB cluster or represents a telencephalon cluster comprising newborn neurons. We have labeled it as Neuron_grm2a+ since we cannot confidently computationally assign a brain region to the cluster beyond generally belonging to the forebrain.”. A glance through the literature indicates that the assumption that tbr1b is a “pallium marker” and is not expressed in the olfactory bulb is not accurate. Daniocell indicates tbr1b is expressed in the olfactory bulb, as do papers in mice (e.g., PMIDs 20807572, 30792833). The other mentioned genes, such as ebf3a have known roles in olfactory bulb development as well (e.g., 14993187). Again, I don’t think that “ebf3a” is a “newborn neuron” marker in general, and its expression does not preclude this cluster from being the olfactory bulb. Much of the provided evidence for cluster annotations in the new Supplementary Table, while helpful, simply cites the senior author’s previous annotations, which are not necessarily a gold standard. I still worry that it can mislead the field because they may not be completely accurate, even though they are now much improved.

© 2024. Published by The Company of Biologists under the terms of the Creative Commons Attribution License (https://creativecommons.org/licenses/by/4.0/).

Regardless, the annotation of these clusters is not the point of the paper. The authors have done a great job responding to all reviewer concerns regarding the tool, which is the point. I would feel more comfortable if a sentence could be added to remind people that these annotations could still be improved with additional refinement by the field or by experts in a certain brain area and that a comprehensive atlas is not the point of this particular paper. Now that this Shiny App is included, however, I am satisfied that the authors have done everything necessary to publish, even if the authors would prefer to not include such a sentence.

2) I appreciate the point about notch being expressed in the xanthophore lineage. I am still confused by the editing in the “erythrocytes” cluster, as those cells do not show any signal on Daniocell. I don’t think this means that the tool is not working as expected. Other unexpected cell types such as pericytes are discussed in the manuscript (and now xanthophores). I would appreciate if a sentence or so was added to give some explanation for the blood cells (previously mentioned in my reviewer comment).

© 2024. Published by The Company of Biologists under the terms of the Creative Commons Attribution License (https://creativecommons.org/licenses/by/4.0/).
